# TRIM21 induces selective autophagy of viruses and bacteria

**DOI:** 10.1016/j.molcel.2026.04.031

**Published:** 2026-06-04

**Authors:** Tyler Rhinesmith, Anna Albecka, Marina Vaysburd, Claudia Puri, Jakub Luptak, Jerome Boulanger, Quynh-Mai Nguyen Le, Matthew J. Gratian, Kevin O’Connell, Lara Few, Mark Donaldson-Wing, Patrycja Kozik, David C. Rubinsztein, and Leo C. James

**Affiliations:** 1 MRC Laboratory of Molecular Biology, Cambridge, UK; 2 Cambridge Institute for Medical Research, Cambridge, UK; 3 UK Dementia Research Institute, Cambridge, UK

## Abstract

TRIM21 is an exceptionally versatile ubiquitin ligase that can be directed by antibodies to target oligomeric protein scaffolds, viral capsids, and proteopathic aggregates for intracellular degradation. How the cell degrades these typically resistant substrates remains poorly understood. To address this, we used TRIM21 viral restriction to create a genome-wide phenotypic screen for antibody-dependent capsid degradation. We identify an antimicrobial selective macroautophagy pathway in mammalian cells, which we term “antibody-directed xenophagy” (ADX). We show that this mechanism restricts structurally diverse pathogens, including adenovirus and *Salmonella*. Using quantitative microscopy, we demonstrate that TRIM21 rapidly intercepts antibody-pathogen complexes, leading to ubiquitin ligase activation. Following this, selective autophagy adaptors are recruited, and viral cargoes are delivered to lysosomes. This process reduces *Salmonella* pathology and bacterial tissue invasion in mice. We propose that TRIM21 evolved through competition with pathogens to induce autophagy of diverse and complex substrates, potentially explaining its versatility for targeted protein degradation.

## Graphical abstract

**Graphical abstract:**
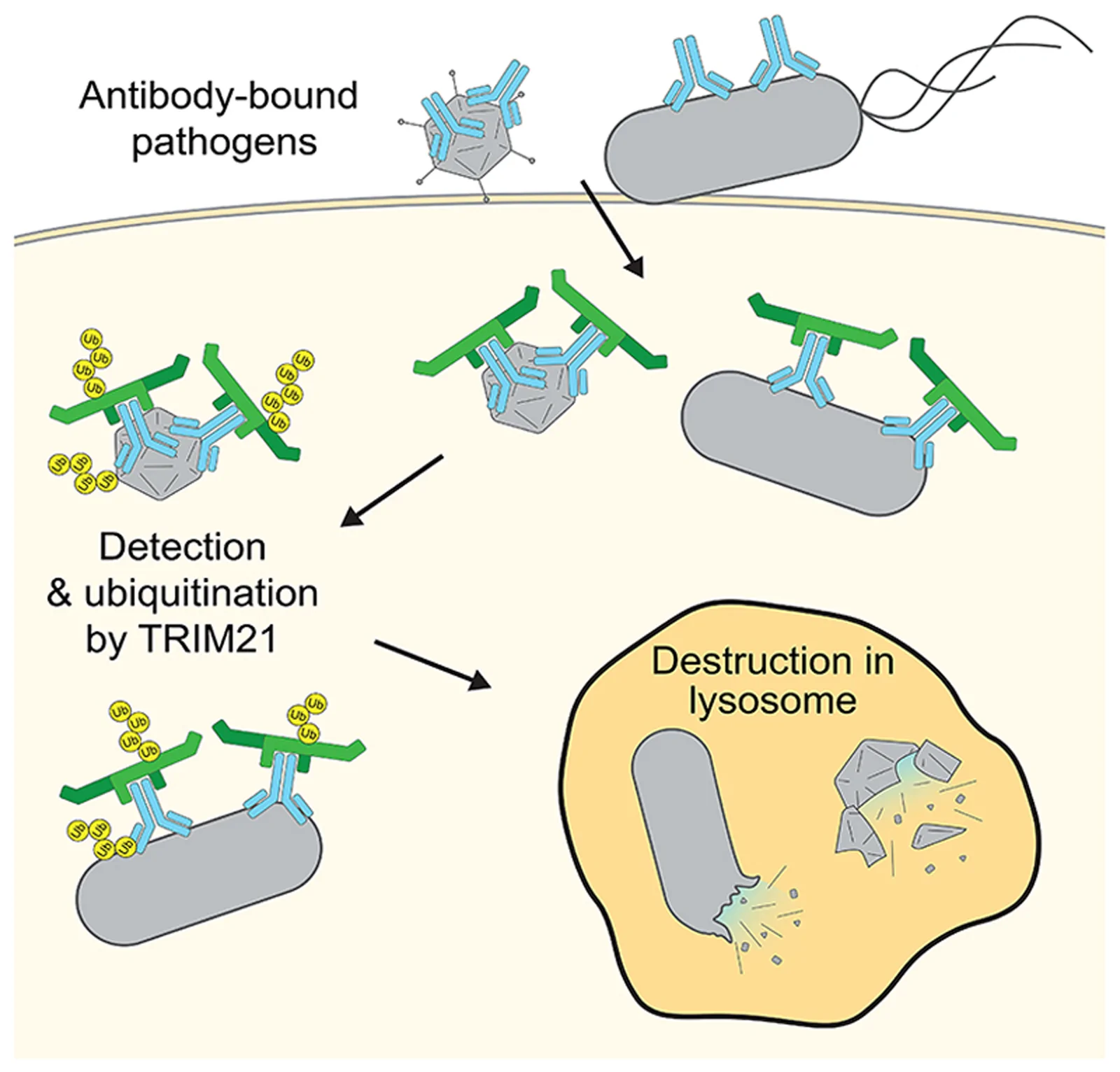

## Introduction

The E3 ubiquitin ligase TRIM21 is an intracellular antibody receptor that binds to antibody-coated viruses in the cytosol and mediates their degradation.^1^ TRIM21 is activated by dimerization of its RING domains, which is driven by clustering of multiple TRIM21 molecules around a substrate.^2,3^ This combination of antibody-directed binding and clustering-based activation enables TRIM21 to restrict phylogenetically and structurally diverse viruses. Recently, we and others have shown that the degradative adaptability of TRIM21 extends to a wide range of additional substrates beyond viral capsid proteins. In the targeted protein degradation (TPD) technology Trim-Away, cytosolic administration of antibodies allows TRIM21 to induce the degradation of a very broad range of neosubstrates, including small soluble proteins and large membrane-bound or nucleic acid-bound complexes.^4^ The capacity for TRIM21 to target challenging substrates is exemplified by its ability to efficiently degrade fibrillar tau, a substrate that normally subverts cellular clearance mechanisms.^5–7^ This has translational importance, as TRIM21-based tau immunotherapy and genetic degraders can prevent tauopathy *in vivo*.^6,8^ In another example of its versatility, TRIM21 can be targeted to degrade viral nucleoproteins, which form very large and highly multivalent complexes with viral nucleic acids. Despite the stability of nucleoproteins, Trim-Away is efficient and can restrict Crimean-Congo hemorrhagic fever virus, lymphocytic choriomeningitis virus, and coronaviruses.^9–11^ These qualities have been leveraged to develop small-molecule compounds that selectively degrade oligomeric protein forms through induced proximity to TRIM21.^12–15^ TRIM21 is thus an important and unique enzyme in the expanding repertoire of E3 ligases used for TPD.^16^ The versatility of its E3 ligase function is enabled because TRIM21 dynamically assembles around its substrate to form enzymatically active oligomeric complexes.^2^ This mechanism of activation is distinct from the widely studied Cullin RING ligases, which must build their substrates into sterically constrained active sites.^17^ While substrate-induced clustering explains how TRIM21 activity can be triggered by structurally diverse targets, it remains unclear how the degradation of complex substrates is actually achieved following TRIM21 activation.

One of the physiological substrates of TRIM21 is the adenovirus capsid, a highly stable icosahedral protein shell that shields viral DNA from cytosolic nucleic acid sensing during the early stages of infection.^18^ Normally, adenovirus controls degradation of its capsid to promote disassembly at the nuclear envelope for successful infection.^19^ Therefore, TRIM21 must intercept and destroy the capsid just after cytosolic invasion and before it reaches the nucleus. We have previously shown that antibodies are carried into the cytosol by invading adenoviruses, and this provokes viral restriction through degradation of the viral capsid in a manner which depends on TRIM21.^3,20^ Nevertheless, how such a large and complex substrate is quickly and efficiently degraded remains unclear. To address this, we used whole-genome CRISPR-Cas9 screening and quantitative imaging to dissect the process of intracellular neutralization by TRIM21. Here, we report that antibodies and TRIM21 trigger selective macroautophagy of adenovirus in a process we term “antibody-directed xenophagy” (ADX). We define the molecular pathway of ADX in high spatial and temporal resolution. We then show that this pathway is pan-specific; in addition to its antiviral activity, ADX also protects against pathogenic *Salmonella* infection *in vitro* and *in vivo*. We postulate that the ability of TRIM21 to trigger selective autophagy of structurally diverse pathogens may underlie the ligase’s broad usefulness for TPD.

## Results

### The autophagy pathway is necessary for antibody-dependent intracellular restriction of Ad5

**Figure 1:**
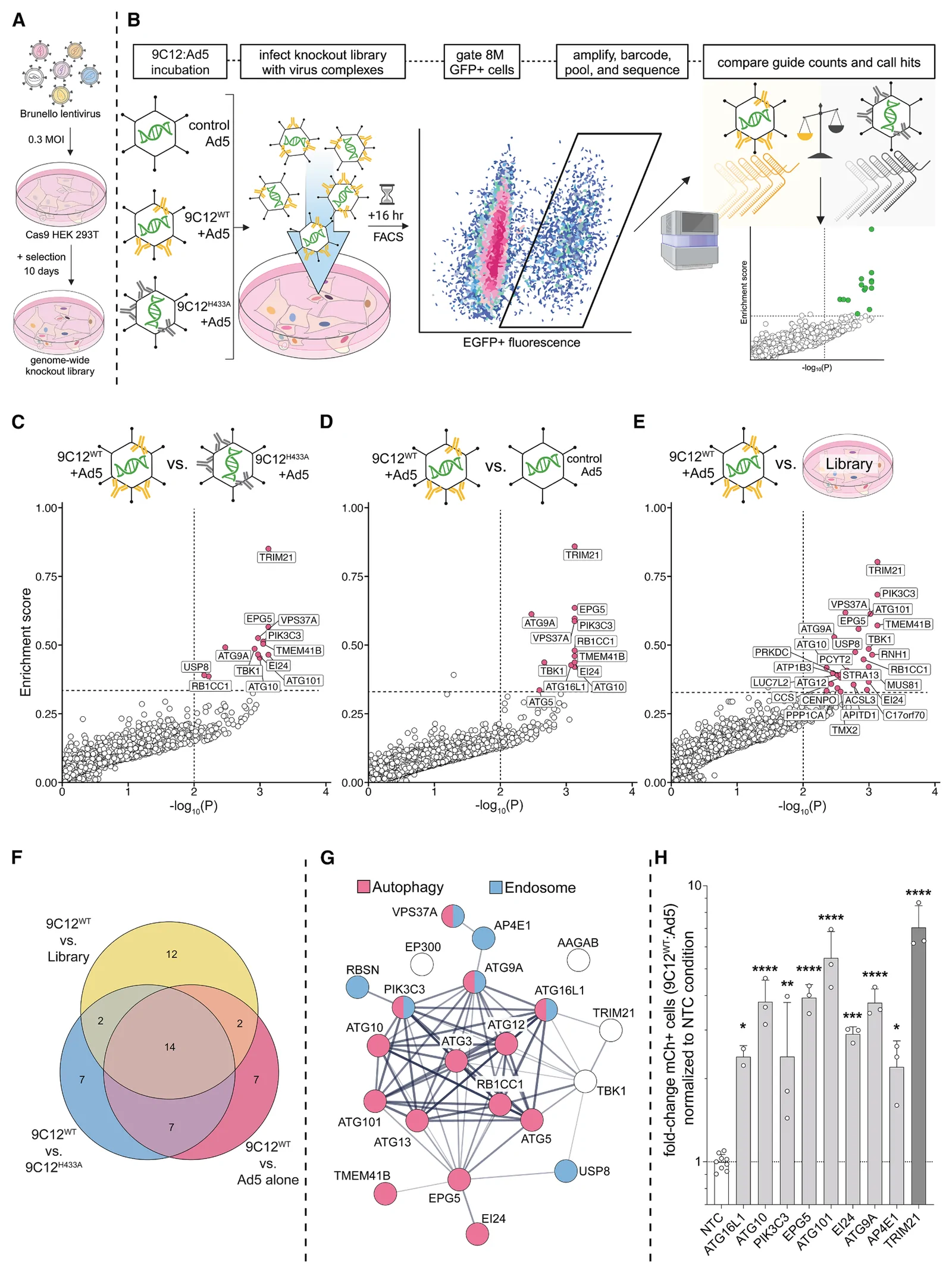
The autophagy pathway is necessary for antibody-dependent intracellular restriction of Ad5 (A) Schematic of lentiviral transduction of Brunello whole-genome library into HEK293T-Cas9 cells. (B) Schematic diagram of genome-wide screen for effectors of antibody-directed intracellular immunity to Ad5. Ad5 was incubated with 9C12^WT^, 9C12^H433A^, or PBS and inoculated on HEK293T-Cas9 cells expressing the Brunello library. EGFP-positive cells were then enriched by fluorescence-activated cell sorting (FACS), and sgRNA sequences were extracted. Effectors of intracellular neutralization by antibodies were identified by comparing sgRNAs enriched in 9C12^WT^ condition with other groups. (C–E) Enrichment of sgRNAs targeting the indicated genes in cells infected with Ad5 incubated with 9C12^WT^ compared with (C) Ad5 incubated with 9C12^H433A^, (D) Ad5 incubated with PBS, or (E) the unsorted library. *n* = 3 biological replicates (*n* = 2 for unsorted library collection). (F) Euler diagram representing overlaps in gene identifications between the indicated comparisons. (G) Functional association network of high-confidence effectors of cytosolic antibody-directed immunity to Ad5. Nodes are colored by selected GO: molecular function family and edge density indicate STRING interaction score. (H) Degree of 9C12^WT^:Ad5 infection in HEK293T-Cas9 cells transduced with indicated sgRNAs. Significance of effect was estimated from *n* = 3 replicates against non-targeting control (NTC) cells by one-way ANOVA with Dunnett’s test for multiple comparisons. Error bars indicate SEM. Asterisks indicate *p < 0.05, **p < 0.01, ***p < 0.001, and ****p < 0.0001.

To identify cellular co-factors of antibody-dependent intracellular viral restriction, we screened a genome-wide knockout (KO) library in HEK293T Cas9 cells (Figures 1A and S1A) with human adenovirus serotype 5 ΔE1ΔE3 harboring enhanced green fluorescent protein (EGFP) (Ad5) complexed to the anti-capsid humanized monoclonal immunoglobulin G (IgG) 9C12 (the complex is notated here as 9C12:Ad5). 9C12^WT^ provokes cytosolic restriction of Ad5 through clustering-induced activation of TRIM21 and capsid degradation.^3,21,22^ This is prevented with 9C12^H433A^, which reduces affinity between TRIM21 and IgG Fc (Figures S1B and S1C).^23^ We reasoned that screening for EGFP+ cells after 9C12^WT^:Ad5 infection would enrich for single-guide RNAs (sgRNAs) targeting effectors of TRIM21 antiviral function (Figure 1B).

We calculated gene-level enrichment scores for sgRNAs from EGFP+ populations from 9C12^WT^:Ad5 infections compared with those from 9C12^H433A^:Ad5 (Figure 1C), Ad5-alone (Figure 1D), or the unsorted library (Figure 1E). All gene-level enrichment data can be found in Table S1. Guide RNAs targeting *TRIM21* were most strongly enriched across these comparisons, consistent with the highly specific nature of IgG-directed capsid degradation by TRIM21. We observed a high degree of overlap between the top 30 enriched sgRNA target genes from each of these three comparisons (Figure 1F). Notably, 70% of the top 30 gene identifications were shared among comparisons in Figures 1C and 1D, while genes identified in the comparison to unsorted library diverged. We next pooled all unique genes in the top 30 lists (51 genes in total), performed functional enrichment analysis (Figure S1D), and then clustered them by association (Figure S1E). Many of the identified genes function in autophagy and endomembrane sorting, while a smaller number are associated with the Fanconi anemia (FA) DNA repair pathway. These latter genes were identified primarily through the enrichment analysis against the unsorted library (Figure 1E), and we considered that their activity might be independent of cytosolic restriction by TRIM21. To test this, we compared the unsorted library with Ad5 infection in conditions without TRIM21 binding. Among the sgRNAs depleted in these EGFP+ populations, our screen identified previously reported Ad5 host factors *CXADR*,^24^ *ITGB5*,^25,26^ and *MIB1*^27,28^ (Figure S1F). To find potential TRIM21-*independent* cellular restriction factors, we performed functional pathway analysis on top sgRNAs that were enriched in these populations. Among these, we again identified FA pathway genes and other DNA-binding proteins (Figure S1G). While FA genes have been implicated in the restriction of other virus families,^29^ in adenovirus infection, FA proteins have previously only been reported to have a pro-viral function.^30^ This warrants further study.

We sought to focus on 9C12-*dependent* restriction factors, so we filtered our pool of 51 putative hits (Figure S1E) to select those found enriched in both 9C12^WT^/9C12^H433A^ and 9C12^WT^/Ad5-alone comparisons. The resultant functional network represents a high-confidence set of gene products likely to be involved in TRIM21-directed intracellular neutralization (Figure 1G). Consistent with their function in an antibody-independent pathway, FA and other DNA-binding genes dropped out entirely from this filtered selection. Retained genes span the entire macroautophagy pathway,^31^ including those whose products comprise members of the ULK1 and PI3KC3 kinase complexes; regulators of lipid delivery, ATG9A and TMEM41B^32,33^; numerous constituents of the Atg8-family conjugation machinery^34^; the ESCRT protein VPS37A that is important for sealing of phagophore membranes^35^; and two lysosomal tethering factors, RBSN and the autophagy-specific EPG5 (Figure S2A).^36–38^ Notably, we identified FIP200 (*RB1CC1*) and TBK1, which have central roles in selective autophagy of pathogens (xenophagy).^39–42^ Although autophagy adapters do not appear in the high-confidence list in Figure 1G, sgRNAs targeting NDP52 (*CALCOCO2*) appear as the 28th-highest enriched pool in the primary screen comparison in Figure 1D. Guide RNAs targeting genes involved in AP-4 transmembrane protein sorting (*AP4E1* and its chaperone *AAGAB*) were also enriched, and this machinery has been shown to influence sub-cellular localization of lipid-bound ATG9A pools.^43–45^ Less well-studied autophagy-linked gene products were also identified, including the deubiquitinase USP8,^46,47^ acetyltransferase EP300,^48^ and the poorly characterized protein EI24.^38^

To validate our findings, we selected genes from this high-confidence list and stably depleted these by using CRISPR-Cas9. We found that restriction of 9C12^WT^:Ad5 was lost in most cell lines tested (Figure 1H). The degree of Ad5 rescue was comparable to depletion of TRIM21, although for a few selected genes, the effect was small or absent (Figure S2B). Consistent with an antibody-specific process, depletion of autophagy genes had no effect on control Ad5 infection (Figure S2C).

### TRIM21 rapidly intercepts cell-invading virions

**Figure 2:**
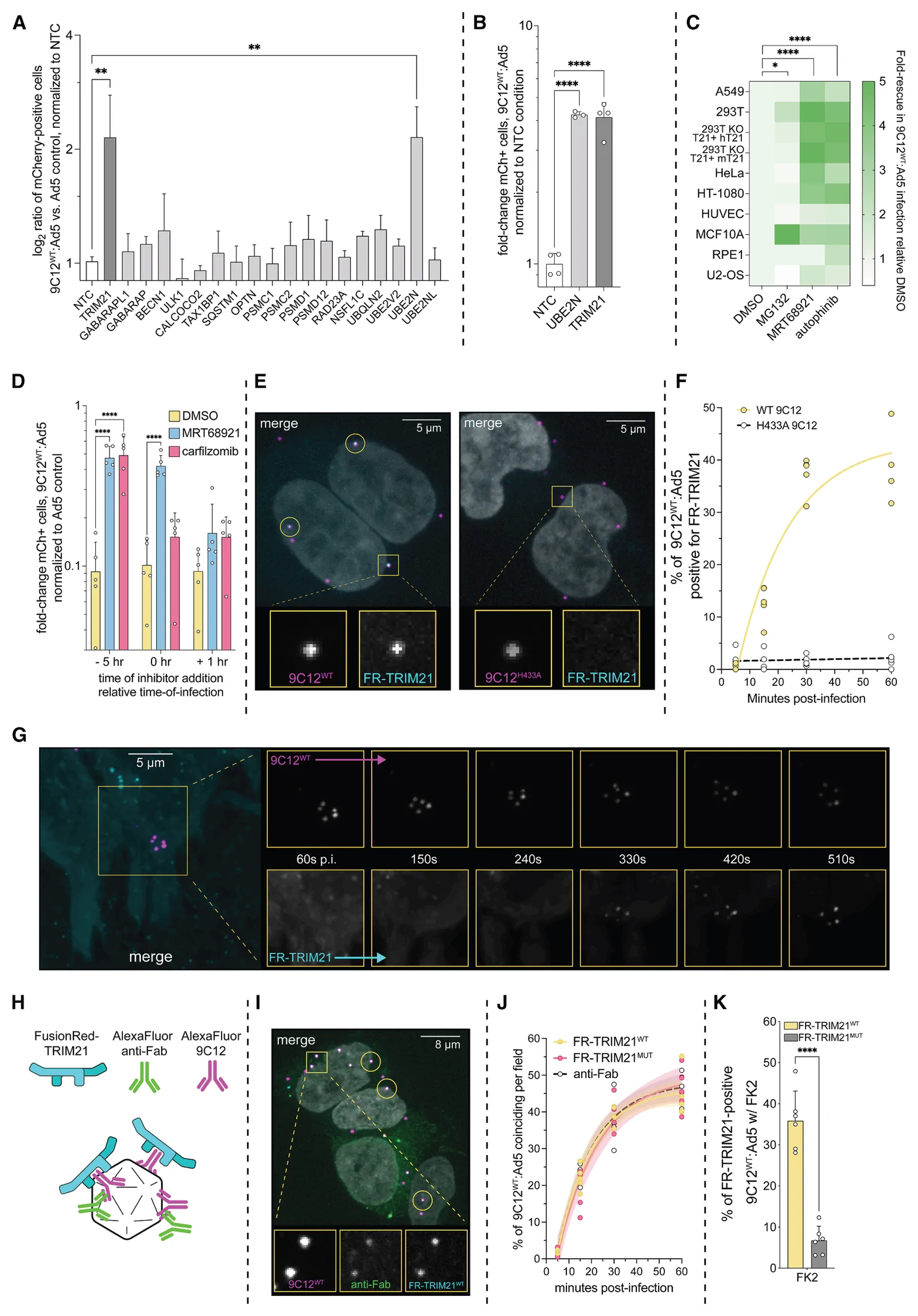
TRIM21 rapidly intercepts cell-invading virions (A) Targeted counter-screen for effectors of antibody-directed intracellular immunity. Indicated genes were transiently depleted in HEK293T, and cells were then infected with Ad5-mCherry complexed with 9C12^WT^. Data are from three separate experiments, with two sgRNAs pooled per gene. Significance of effect was estimated against NTCs by one-way ANOVA with Dunnett’s post hoc test. (B) 9C12^WT^:Ad5 infection in HEK293T-Cas9 cells transduced with indicated sgRNAs. Significance of effect was estimated from *n* = 3 biological replicates against NTC by one-way ANOVA with Dunnett’s test. (C) Panel of cells pre-treated for 2 h with the indicated inhibitors and infected with 9C12^WT^:Ad5. Green cells were counted, normalized to confluency, and plotted relative to the DMSO control condition. Treatment-wise significance was estimated by two-way ANOVA corrected for multiple comparisons using Tukey’s test. *n* = 2 biological replicates for each cell line. (D) Time-of-addition experiment for inhibitors of the proteasome and ULK1/2. Cells were treated with MG132 or MRT68921 at the indicated time points relative to time of infection with 9C12^WT^:Ad5. Significance was estimated by two-way ANOVA with Dunnett’s test. Data are from five independent experiments. (E–G) Co-localization of FR-TRIM21 with AF647-9C12^WT^:Ad5, (E) in cells fixed 30 min following synchronized infection, (F) quantified at intervals over 1 h, and (G) occurring rapidly in live cells. Quantifications are from *n* = 4–5 fields per time point. (H–J) Cells expressing (H) FR-TRIM21 were electroporated with anti-Fab-488, infected with Ad5 complexed with AF647-9C12^WT^, and co-localization was detected using confocal microscopy. (I) Representative image at 30 min following synchronized infection. (J) Co-localization quantified at intervals over 1 h post-infection, from *n* = 4–5 fields per time point. Filled regions represent 95% confidence intervals. (K) Quantification of AF-9C12^WT^:Ad5 co-localized with FR-TRIM21^WT^ or FR-TRIM21^MUT^ and FK2 puncta 20 min following synchronized infection. Significance was estimated by unpaired two-tailed *t* test. Quantifications from *n* = 6 image fields per time point. Error bars indicate SEM. Asterisks indicate *p < 0.05, **p < 0.01, ***p < 0.001, and ****p < 0.0001.

We previously demonstrated that intracellular antibodies restrict Ad5 by driving TRIM21 clustering on the opsonized viral capsid, which promotes RING dimerization.^2,20^ Inhibitor treatment experiments suggested that viral infection was terminated by degradation of the capsid in the cytosol, mediated by the 26S proteasome.^3^ In our genome-wide screen, guide RNAs targeting components of the proteasome were depleted from the library (Table S2), so we could not directly interrogate this model in the primary screen. Instead, we used transient knockdowns in a targeted counter-screen to compare hits from our primary screen with a hypothesis-driven list of test genes. These comprised proteasome subunits, ubiquitin-binding shuttles, E2 ubiquitin-conjugating enzymes, and autophagy-related genes that did not enrich in the primary screen—including selective autophagy adaptors. Taken as a whole, transient knockdown of genes identified in the primary screen rescued 9C12^WT^:Ad5 infection, while hypothesis-driven selections did not (Figure S3A). Consistent with previous reports showing that TRIM21 can catalyze formation of K63 polyubiquitin chains, knockdown of the K63-specific E2 ubiquitin-conjugating enzyme UBE2N rescued 9C12^WT^:Ad5 infection^49,50^ (Figure 2A). We confirmed this phenotype in stable UBE2N KO HEK293T cells (Figures 2B and S3B). KO of UBE2N affected 9C12^WT^:Ad5 restriction to an extent similar in magnitude to KO of autophagy genes (Figures S3B and S3C). No additional rescue of 9C12^WT^:Ad5 infection occurred when any of these genes were depleted in TRIM21 KO cells (Figure S3D). Some TRIM proteins have been described as autophagy regulators,^51^ so we also tested whether TRIM21 depletion affected steady-state LC3 lipidation in HEK293T cells (Figure S3E). We detected no differences in autophagic flux in TRIM21 KO cells, and this was not altered by infection with Ad5 or 9C12^WT^:Ad5. It is therefore probable that the small size of the virus and the low multiplicity of infection (∼1 virion per 3 cells) do not measurably affect whole-cell flux through autophagy. We concluded that TRIM21, UBE2N, and autophagy effectors operate on a single pathway coordinated by intracellular antibodies to specifically restrict cytosolic virions.

We next used pharmacological inhibitors of the 26S proteasome or autophagy to test cytosolic neutralization of Ad5 across a range of human cell lines. While 2-h pre-treatment with inhibitors had a minimal effect on Ad5 infection without antibodies (Figure S3F), both inhibitor classes rescued infection in the presence of 9C12^WT^ (Figure 2C). With 1 h of pre-treatment, inhibition of autophagy initiation (MRT68921^52^; autophinib^53^) restored 9C12^WT^:Ad5 infectivity to a greater extent than the proteasome inhibitor MG132. We next compared the effect of inhibitors added with or without pre-treatment (Figure 2D). While pre-treatment with proteasome inhibitors rescued infection, their addition at the time of infection did not. Indeed, the degree of infection rescue scaled with the duration of proteasome inhibitor pre-treatment and the required 7-h pre-incubation in HEK293T for maximal effect (Figure S3G). Meanwhile, autophagy inhibition with MRT68921 did not require pre-treatment to rescue infection; addition of autophagy inhibitors at the time of infection was as effective as pre-treatment in HEK293T (Figure 2D) The same phenomenon was observed across a range of human cell lines (Figure S3H). This lag time of effect for the proteasomal pheno-type contrasts with mechanistic studies on proteasome inhibitors, using proteolysis targeting chimera (PROTAC) molecules, where onset of the inhibitory phenotype is essentially instantaneous.^54^ We used a luminescence-based live-cell proteasome activity assay to confirm that the concentrations of proteasome inhibitor used in this study (1–2.5 μM MG132) rapidly and potently inhibited the proteasome (Figure S3I). The proteasome must therefore be profoundly inhibited for hours before infection to observe any impact on TRIM21-mediated neutralization. We next used the proteasome activity assay to confirm that chemical autophagy inhibitors did not affect the proteasome over relevant timescales (Figure S3J). In agreement with this, we detected no additive effect of co-treatment with proteasome and autophagy inhibitors (Figure S3K). Our data are therefore inconsistent with a role for the proteasome in proteolysis of opsonized viruses in the cytosol. Proteasome inhibition may instead indirectly suppress viral autophagy by interfering with polyubiquitin hydrolysis by the proteasome, thereby reducing free ubiquitin availability for TRIM21.^55^ Together, these chemical inhibition studies align with results from the genome-wide screens that autophagy, and not the proteasome, is induced by TRIM21 to restrict antibody-opsonized cytosolic viral particles.

Importantly, addition of MRT68921 at the time of infection was sufficient to rescue 9C12^WT^:Ad5 infectivity, and pre-treatment did not enhance its effect. However, addition of MRT68921 at 1 h following infection was ineffective (Figure 2D). Therefore, we hypothesized that TRIM21 viral neutralization hinges on a critical series of molecular events that must occur in a 1-h window following infection. To study this time frame, we used confocal microscopy to track Ad5 infection at high spatial and temporal resolution. We fused the red fluorescent protein^56^ FusionRed^MQV^ to wild-type (WT) TRIM21 (FR-TRIM21^WT^) or RING domain mutant TRIM21^I18R/M72E^ (FR-TRIM21^MUT^) and stably expressed them in HEK293T KO TRIM21 cells (Figure S4A). FR-TRIM21^MUT^ contains mutations I18R/M72E, which reduce E2 binding and RING dimerization. Control experiments confirmed that FR-TRIM21^WT^ rescued restriction of Ad5 (Figure S4B), and this depended on RING domain activity. While FR-TRIM21^WT^ was not as potent as the endogenous enzyme, it was sufficiently active to model its function. We also made Alexa Fluor 647-9C12^WT^ or 9C12^H433A^ (AF-9C12^WT/H433A^) antibodies to visualize antibody-bound cytosolic viruses. These modified anti-bodies restricted Ad5 with WT activity (Figure S4C). Consistent with FR-TRIM21 binding to the Fc region of cytosolic 9C12, we observed robust co-localization between FR-TRIM21^WT^ and AF-9C12^WT^:Ad5 after infection, but this was lost with the H433A mutant antibody (Figure 2E). Association between FR-TRIM21 and AF-9C12^WT^:Ad5 puncta occurred as early as 5 min following synchronized infection, reaching a maximum at 30 min and plateauing thereafter (Figure 2F). In live cells, we observed a diffuse FR-TRIM21 signal rapidly coalescing around AF-9C12^WT^:Ad5 following synchronized viral infection (Figure 2G; Video S1; STAR Methods). The kinetics of TRIM21 binding to opsonized Ad5 therefore fit a 1-h window for Ad5 restriction.

It has previously been shown that adenoviruses access the cytosol within minutes following endocytosis,^57^ but the impact 9C12 has on this is unclear. We therefore tested the fraction of 9C12-bound virions that successfully escaped endosomes. We electroporated FR-TRIM21 cells with fluorescently labeled anti-Fab antibodies and then infected these with AF-9C12^WT^:Ad5. We reasoned that any 9C12 that reached the cytosol would become bound by anti-Fab, allowing quantification of cytosolic AF-9C12^WT^:Ad5 (Figure 2H). We readily observed coincidence of 9C12^WT^, anti-Fab, and FR-TRIM21^WT^ (Figure 2I) or FR-TRIM21^MUT^ (Figure S4D). Strikingly, association kinetics between AF-9C12^WT^:Ad5 and anti-Fab or FR-TRIM21 were identical at all time points tested, showing that FR-TRIM21 is a reliable reporter for 9C12^WT^:Ad5 cytosolic penetration (Figure 2J). FR-TRIM21^WT^ and FR-TRIM21^MUT^ co-localized identically with 9C12^WT^:Ad5, consistent with existing data that RING domain mutations do not affect TRIM21-Fc binding but rather inhibit its E3 ligase activity.^2,58^ Indeed, we detected a 5-fold increase in FK2-positive polyubiquitin puncta co-localizing with FR-TRIM21^WT^ over FR-TRIM21^MUT^ (Figures 2K and S4E). To confirm a physical interaction between TRIM21 and opsonized viruses, we developed a split luciferase-based probe for cytosolic complex formation. In 293T cells we co-expressed LgBiT-TRIM21 and SmBiT-TRIM21, which reconstitute luciferase activity only when they bind a shared substrate (Figure S4F).^59^ We also co-expressed LgBiT fused to tandem ubiquitin-binding entities (LgBiT-TUBEs) and SmBiT-TRIM21 to test for polyubiquitin around viral complexes. Upon infection with Ad5 coated in 9C12^WT^, we observed a robust luciferase signal indicating TRIM21 binding and polyubiquitination around virus complexes, and this was lost with the mutant 9C12^H433A^ that does not bind TRIM21. Taken together, results from the genome-wide screen and confocal imaging experiments of TRIM21 indicate that autophagic degradation of antibody-bound Ad5 capsids occurs swiftly after infection and is triggered by TRIM21-mediated polyubiquitination.

### TRIM21 provokes ADX of Ad5

**Figure 3:**
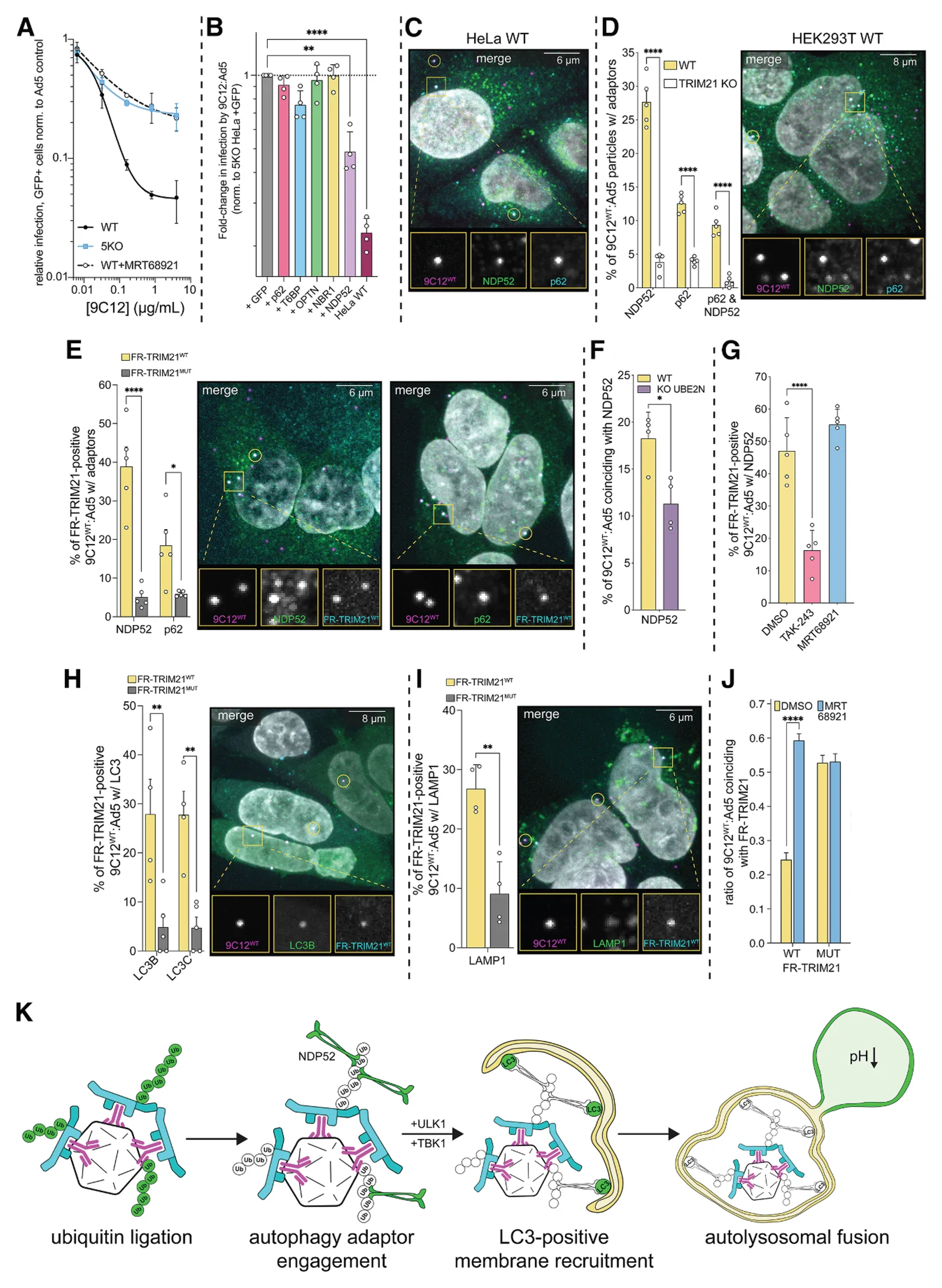
TRIM21 provokes ADX of Ad5 (A) Restriction of Ad5 complexed to indicated amounts of 9C12^WT^ in HeLa 5KO cells or WT cells treated with 1 μM MRT68921. Data from *n* = 3 independent replicates, analyzed for significance by one-way ANOVA with Dunnett’s test. (B) Measurement of 9C12^WT^:Ad5 infection in HeLa 5KO cells complemented with the indicated autophagy adaptor. The fraction of infected cells in each background was normalized to 5KO cells complemented with EGFP alone, and significance was estimated from *n* = 3 independent experiments by one-way ANOVA and Dunnett’s test for multiple comparisons. (C–E) Representative confocal micrographs of co-localization between AF-9C12^WT^:Ad5, NDP52, and p62 at 30 min post-infection in (C) HeLa cells, (D) WT HEK293T, and (E) FR-TRIM21^WT^ cells. For quantifications against control cell lines presented in (D) and (E), we used *n* = 5 fields per condition for two-way ANOVA with Šídák’s test for multiple comparisons. (F and G) Quantification of co-localization between NDP52 and AF-9C12^WT^:Ad5 at 30 min following synchronized infection, in (F) HEK293T cells depleted in UBE2N, compared with control (two-tailed *t* test), and (G) FR-TRIM21^WT^ cells following 5 h pre-treatment with the indicated compounds (one-way ANOVA and Benjamini-Krieger-Yekutieli procedure). Quantifications were from *n* = 4–5 fields per condition. (H and I) Representative confocal micrographs and quantifications of AF-9C12^WT^:Ad5 co-localized with FR-TRIM21 and (H) LC3 at 30 min (two-way ANOVA with Séidaé k’s test) or (I) LAMP1 at 45 min, following synchronized infection (two-tailed *t* test). Quantifications were from *n* = 4 fields per condition. (J) Per-cell quantification of FR-TRIM21^WT^ or FR-TRIM21^MUT^ puncta co-localizing with AF-9C12^WT^:Ad5 at 2 h post-infection, with and without MRT68921. Significance was estimated via two-way ANOVA with Tukey’s test. Quantifications were from *n* = 5 image fields per condition. (K) Model for antibody-directed cytosolic neutralization of Ad5 by selective autophagy. TRIM21 clustering around opsonized virions in the cytosol activates its E3 ligase activity, which signals for autophagy adaptor recruitment. This drives nucleation of the phagophore and incarceration of the virion, which is eventually delivered to lysosomes for degradation. Error bars indicate SEM. Asterisks indicate *p < 0.05, **p < 0.01, ***p < 0.001, and ****p < 0.0001.

Antiviral selective autophagy (xenophagy) is poorly characterized,^60^ so we next dissected the mechanism of TRIM21-mediated Ad5 xenophagy. We first tested whether ubiquitin-binding selective autophagy adaptors are required for restriction. Simultaneous KO of p62, NBR1, NDP52, optineurin, and TAX1BP1 in HeLa cells^61^ (HeLa-5KO) phenocopied autophagy inhibition by MRT68921 (Figure 3A). We individually reconstituted each adaptor (Figure S5A) in HeLa-5KO and found that NDP52 partially rescued 9C12^WT^:Ad5 infection (Figure 3B) but had no effect on Ad5 infection without antibodies (Figure S5B). In confocal microscopy experiments using WT HeLa cells, AF-9C12^WT^:Ad5 co-localized with NDP52 and p62 (Figure 3C), but AF-9C12^H433A^:Ad5 did not (Figure S5C). Similarly, co-localization between autophagy adaptors and AF-9C12^WT^:Ad5 was substantially reduced in TRIM21-deficient HEK293T compared with WT (Figure 3D). This could be rescued by expression of FR-TRIM21^WT^ but not catalytically inactive FR-TRIM21^MUT^ (Figure 3E), demonstrating that autophagy adaptor recruitment is dependent on TRIM21 antibody binding and ubiquitination activity. In HEK293T cells that expressed endogenous TRIM21 but were deficient in UBE2N, we detected a reduction in total AF-9C12^WT^:Ad5 co-localizing with NDP52, compared with WT cells (Figure 3F). This is consistent with reports that K63-linked polyubiquitin regulates autophagy.^62,63^ The inhibitor of E1 ubiquitin-activating enzyme TAK-243^64^ also reduced NDP52 coincidence with AF-9C12^WT^:Ad5 particles positive for FR-TRIM21^WT^, further confirming ubiquitin-dependent selective autophagy adaptor recruitment (Figures 3G and S5D). Notably, inhibition of ULK1/2 and TBK1 with MRT68921 had no impact on NDP52 association, placing adaptor recruitment upstream of autophagy activation machinery.

Since our primary screen detected loss-of-function phenotypes related to genes involved in Atg8-family conjugation and autolysosomal fusion, we also investigated these processes using our confocal imaging system. AF-9C12^WT^:Ad5- and FR-TRIM21^WT^-positive puncta co-localized with LC3B and the xenophagy-specific LC3C (Figure 3H), and these were reduced in cells expressing FR-TRIM21^MUT^. Similar results were obtained when we co-stained for lysosome-associated membrane protein 1 (LAMP1; Figure 3I). We next used structured illumination super-resolution microscopy to visualize the morphology of LC3-positive autophagosomes^65,66^ in FR-TRIM21^WT^ cells infected with antibody-coated adenovirus (Figure S5E). 9C12^WT^-bound adenoviruses, which co-localized with FR-TRIM21, were much more likely to be found in LC3 compartments than naked viruses. LC3 formed hollow globular compartments that enveloped these FR-TRIM21 and 9C12^WT^ complexes. When we treated cells with ammonium chloride, which stalls autophagosome closure, TRIM21-postive viruses became trapped in very large LC3-positive compartments. Using confocal microscopy, we noted a decrease in co-localization between FR-TRIM21^WT^ and AF-9C12^WT^:Ad5 beginning at 60 min post-infection (Figure S6A) and peaking after 120 min (Figure 3J). This depletion was stabilized in FR-TRIM21^MUT^ cells and with MRT68921 treatment, consistent with TRIM21-mediated, ubiquitin-dependent autophagic degradation of TRIM21:9C12^WT^:Ad5 complexes. In live cells expressing FR-TRIM21^WT^, we observed a loss of the pH-sensitive EGFP-LC3B signal from AF-9C12^WT^:Ad5 puncta that were positive for LysoTracker, a marker for acidic compartments (Figure S6B). Loss of the EGFP-LC3B signal was accompanied by dimming of AF-9C12^WT^ fluorescence and a morphological transition from punctate to diffuse FR-TRIM21. This corresponded with the formation of large LAMP1-positive structures with visible lumens that contained diffuse 9C12^WT^ and FR-TRIM21^WT^ (Figure S6C). These observations were consistent with delivery and degradation of TRIM21- and antibody-coated Ad5 in acidic lysosomes. We next developed a tandem-fluorescent probe to directly test whether 9C12^WT^:Ad5 particles are delivered to acidified compartments. We fused EGFP and FusionRed^MQV^ to a camelid nanobody against the Fab domain of human IgG^67^ and expressed this in HEK293T cells. The anti-Fab nanobody binds to 9C12 at a distinct epitope from TRIM21, and since EGFP is more acid-labile than FusionRed, low pH causes conversion of yellow puncta to red puncta in lysosomes. In line with selective autophagy induced by TRIM21, we detected a trend toward viruses co-localizing with red-only puncta when complexed with 9C12^WT^ but not the H433A mutant, suggesting that the former are indeed delivered to lysosomes (Figure S6D).

To gather direct evidence of viral protein degradation and neutralization by this process, we infected cells with high-titer Ad5, with and without 9C12^WT^ and inhibitors. In cell lysate collected 2 h after infection, the viral structural protein hexon was substantially degraded with 9C12^WT^, and this was not reversed by proteasome inhibition (Figure S6E). Consistent with the imaging-based results, TRIM21 was co-degraded with hexon, and this was stabilized by bafilomycin A1. Hexon levels were higher in bafilomycin A1-treated cells than in the control infection without 9C12, potentially because virions that fail to escape endosomes were no longer being degraded. To demonstrate that TRIM21 targets infectious viral particles through autophagy, we measured *de novo* hexon production 48 h following infection (Figure S6F). As expected, 9C12^WT^ profoundly suppressed viral protein production. In the absence of 9C12, inhibitors did not measurably affect hexon production. Strikingly, when TRIM21 activity was stimulated by 9C12^WT^, only autophagy inhibition rescued hexon protein levels 48 h later. Taken together, all these suggest that TRIM21 ubiquitin ligase activity is required for the incarceration of viral cargoes in autophagosomes and their delivery to lysosomes for degradation. We term this process ADX, depicted in Figure 3K. In ADX, TRIM21 marks antibody-bound viral complexes with polyubiquitin, which recruits autophagy adaptors. LC3-positive membranes are then built around virions, and these are finally trafficked to lysosomes for degradation.

### ADX restricts *Salmonella*

**Figure 4:**
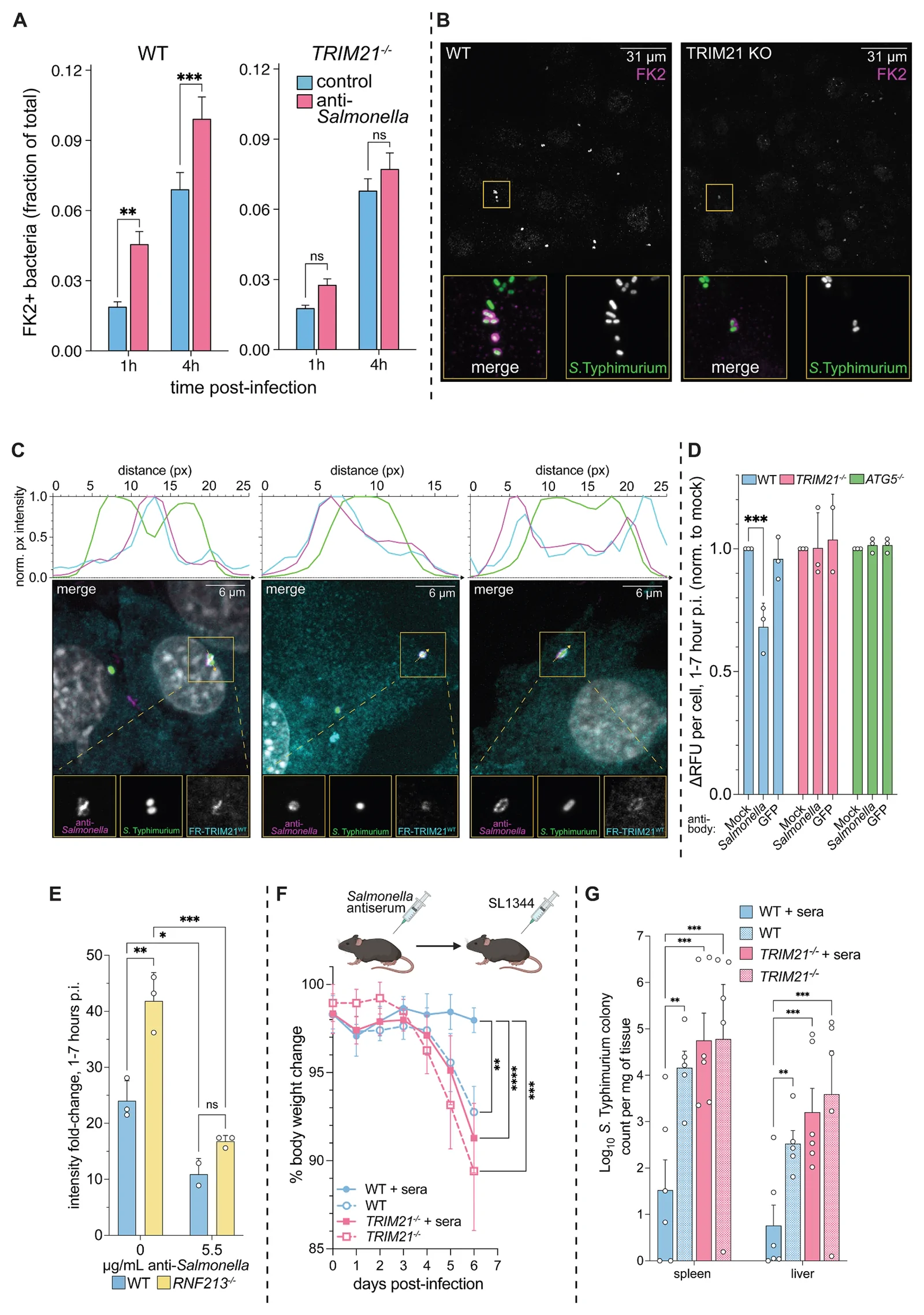
ADX is effective against *Salmonella* (A and B) Polyubiquitin-co-localizing *S*. Typhimurium (A) quantification and (B) representative confocal micrographs (1 h post-infection) in WT and *TRIM21*^−/−^ MEFs inoculated with bacteria pre-treated with anti-*Salmonella* antibodies. Significance was estimated by two-way ANOVA and Tukey’s test for multiple comparisons. Quantifications were from *n* = 20 fields per condition. (C) Representative confocal micrographs of *S*. Typhimurium pre-treated with anti-*Salmonella* antibodies co-localizing with FR-TRIM21^WT^. The normalized fluorescence intensity in each channel along a line profile bisecting selected bacteria is displayed above each merge. (D and E) Per-cell change in fluorescence intensity of infected WT MEFs with cytosolic hyper-proliferating *S*. Typhimurium measured from 1 to 7 h post-infection, incubated with 5.5 μg/mL anti-*Salmonella* antibodies, compared with (D) *TRIM21*^−/−^ and *ATG5*^−/−^ or (E) *RNF213*^−/−^ MEFs. For ease of comparison, data in (D) are normalized to the “mock” condition. Significance was estimated from *n* = 3 experiments by two-way ANOVA with Dunnett’s test (D) or Šídák’s test (E). (F and G) Passive serum transfer experiment in C57BL/6 or *TRIM21*^−/−^ mice. Anti-*Salmonella* antisera or PBS was administered to naive mice, and these were infected with *S*. Typhimurium strain SL1344. (F) Significance of body weight loss was estimated by mixed-effects model and Dunnett test for multiple comparisons. (G) Bacterial tissue load measured by CFU assay with two-way ANOVA and Šídák’s test for multiple comparisons. There were *n* = 6 mice per group. Error bars indicate SEM. Asterisks indicate *p < 0.05, **p < 0.01, ***p < 0.001, and ****p < 0.0001.

Owing to TRIM21’s ability to assemble around a broad range of substrate types to activate its E3 ligase, we hypothesized that the ADX pathway could restrict pathogenic bacteria as well as viruses. To test this, we infected mouse embryonic fibroblasts (MEFs) with the model bacterial pathogen *Salmonella enterica* serovar Typhimurium (*S*. Typhimurium) and performed FK2 poly-ubiquitin staining and confocal microscopy. We detected an increased fraction of ubiquitin-positive bacteria at 1 and 4 h post-infection with anti-*Salmonella* antibodies, and this was dependent on TRIM21 expression (Figure 4A). Furthermore, an analysis of thousands of individually quantified ubiquitin-positive bacteria revealed that antibody treatment was associated with more intense FK2 staining in WT but not TRIM21 ^−/−^ cells at 1 h post-infection (Figures 4B and S7A). While predominantly vacuolar, *S*. Typhimurium perforates the vacuole and becomes cytosolic in ∼ 10% of infected cells, which can result in cytosolic exposure of antibodies.^49,68,69^ We found that FR-TRIM21 co-localized with anti-*Salmonella* antibodies around the perimeters of antibody-opsonized bacteria, confirming that these *Salmonella* are cytosolic and bound by TRIM21 (Figure 4C). Similar results were obtained from MEFs electroporated with anti-*Salmonella* antibodies prior to infection (Figure S7B). In this case, where only cytosolic bacteria could be bound by the pre-electroporated antibodies, twice as many antibody-bound bacteria were polyubiquitin-positive in WT compared with TRIM21^™/™^ MEFs (Figure S7C). We hypothesized that more prevalent and robust polyubiquitin coats on antibody-opsonized bacteria in the cytosol might promote autophagy-dependent clearance.^70^

Once *S*. Typhimurium escapes into the cytosol, it hyper-proliferates—dividing far more rapidly than vacuolar bacteria—and hyper-proliferation can serve as a marker for cytosolic penetration.^71,72^ We anticipated that anti-*Salmonella* antibodies would block cell invasion through extracellular neutralization, and this effect would need to be separated from intracellular ADX.^69^ We therefore developed a wide-field imaging-based technique to quantify the hyper-proliferation of fluorescent bacteria in live infected MEFs (Figure S7D). We measured the real-time fluorescence intensity of hyper-proliferating (cytosolic) bacteria at single-cell resolution (Figure S7E) and calculated the relative rate of change during log phase intracellular growth (Figure 4D). Pre-incubation with anti-*Salmonella* antibodies attenuated the growth rate of hyper-proliferating *S*. Typhimurium in MEF^WT^ but not *TRIM21* ^−/−^ or *ATG5* ^−/−^ cells. This effect was specific to antibodies against *Salmonella* (Figure S7F). Our imaging approach allowed us to measure antibody-dependent entry blocking separately from intracellular antiproliferative effects for the first time^69^ (Figure S7G). At very high antibody densities, too few bacteria successfully invaded cells to quantify hyper-proliferation. To confirm that the intracellular antiproliferative effect was antibody-specific, we diluted the bacterial inoculum to levels below those achieved with antibodies, and this did not reduce the rate of hyper-proliferation observed in infected cells (Figure S7H). Thus, in addition to classical extracellular neutralization, whereby opsonization reduces cell penetration by bacteria, antibodies also exert a protective effect in the cytosol in a manner analogous to ADX of non-enveloped viruses. Recently the E3 ligase RNF213 was shown to recognize and ubiquitinate *S*. Typhimurium, leading to its restriction by selective autophagy.^73^ We therefore sought to test the interplay between RNF213 and ADX of *Salmonella*. As previously reported, cytosolic *S*. Typhimurium proliferated more rapidly in *RNF213* ^−/−^ cells compared with WT. Strikingly, this accelerated growth was completely attenuated when bacteria were pretreated with anti-*Salmonella* antibodies (Figure 4E), an effect that depended on *TRIM21* (Figure S8A). Conversely, RNF213 could not fully compensate for a lack of antibody-mediated activity. Taken together, our data suggest that TRIM21 and RNF213 antibacterial activities are complementary, with the strongest block to proliferation observed in cells that express both ligases and are treated with antibodies.

To directly measure TRIM21 binding to antibody-coated bacteria, we used a split-nanoluciferase approach similar to that described above. We mixed recombinant SmBit-TRIM21 and LgBiT-TRIM21 proteins with anti-lipopolysaccharide (LPS) antibody and *S*. Typhimurium (Figure S8B). Luminescence was reconstituted when antibodies clustered TRIM21 proteins around the bacterial coat but not in conditions without *Salmonella*. This result was consistent with previous work demonstrating that substrates trigger TRIM21 activity through antibody-directed clustering.^2^ We next tested whether TRIM21 activation leads to *Salmonella* restriction through the ADX pathway by staining cells for p62. We found that antibody-coated bacteria co-localized with p62 to a greater extent in cells that expressed TRIM21 (Figure S8C). Treatment with antibodies rescued *Salmonella*-induced lipidation of LC3 in infected cells, likely a result of restriction by TRIM21 and suppression of secreted bacterial effectors^74^ (Figure S8D). In addition, super-resolution microscopy confirmed that MEFs lacking TRIM21 have a profound defect in the formation of LC3 coats around antibody-opsonized bacteria in the cytosol, supporting our hypothesis that TRIM21 is responsible for triggering ADX of *Salmonella* (Figure S8E).

Finally, to demonstrate the physiological importance of these findings, we compared *S*. Typhimurium infection in C57BL/6 WT and *TRIM21* ^−/−^ mice in the presence and absence of anti-*Salmonella* serum. Cell-free immune serum from donor animals inoculated with a vaccine strain of *S*. Typhimurium was administered to naive mice, which were then infected with pathogenic *S*. Typhimurium strain SL1344. Transferred antiserum prevented disease-associated weight loss in WT but not *TRIM21* ^−/−^ mice (Figure 4F). Following tissue collection, we quantified bacterial organ load in infected animals, using a standard colony-forming unit (CFU) assay. Colonization of spleen and liver was ∼ 100-fold less in WT animals administered serum than PBS, and this protection was lost in *TRIM21* ^−/−^ mice (Figure 4G). Similar results were obtained when we quantified tissue-extracted bacterial DNA by PCR (Figure S8F). Thus, TRIM21 protects against animal models of bacterial infection, extending its known antiviral role *in vivo*.^10,75^

## Discussion

Here, we report that the E3 ligase TRIM21 mediates a system of ADX, which protects cells from infection by rapidly targeting incoming viruses and bacteria for lysosomal degradation. Intracellular ADX bears a conceptual resemblance to antibody-stimulated phagocytosis of opsonized pathogens and infected cells. As with membrane-bound FcRs expressed on phagocytes, TRIM21 activity is triggered by antibody-induced clustering around immune complexes.^2,76^ TRIM21 activation then drives autophagy, a cytosolic process analogous to phagocytosis. The incarceration and degradation of pathogens in acidified membrane-bound compartments thus represents a conserved effector function of immunoglobulins in multiple contexts. By linking antibodies to cytosolic antimicrobial signaling, ADX bridges humoral immunity with cell-autonomous innate immunity. However, the adaptive nature of antibodies contrasts ADX with other mechanisms of cell-autonomous immunity driven by the direct recognition of pathogen-associated molecular patterns (PAMPs). For example, RNF213 senses LPS signatures on some bacteria to trigger their ubiquitination and autophagic degradation.^73^ We show that TRIM21 and RNF213 provide complementary and non-overlapping levels of protection against *S. Typhimurium*. While RNF213 provides strong immunity, we find that TRIM21 and ADX are required for full protection and can rescue RNF213 deficiency. Because the antibody response can rapidly adapt to pathogen evolution, we postulate that TRIM21-mediated ADX could compensate for bacterial evasion of RNF213 through bacterial LPS modification.

Cell-autonomous sensing of viruses is more challenging than that of bacteria, as viruses possess fewer unique metabolites and molecules that distinguish them from self. Instead, cell-autonomous sensing of viruses is reliant on the identification of more subtle molecular patterns of infection, such as unique protein and nucleic acid signatures or mis-localized self molecules.^77^ Unlike the conserved mechanisms reported for antibacterial xenophagy,^78^ there are many bespoke processes for antiviral xenophagy,^29,79,80^ including some pathways involving TRIM-family proteins.^51,81,82^ These sometimes only function in particular tissue contexts,^83,84^ and many mechanisms remain contested^85–87^ perhaps because viruses carry factors that interfere with or hijack antiviral xenophagy.^60,88,89^ Some, such as hepatitis C virus, may shut off autophagy during viral entry, only to upregulate it during replication.^90^ These considerations have made studying antiviral xenophagy particularly challenging. In contrast, ADX is an exquisitely inducible, generalist mechanism of antiviral immunity. It functions in many mammalian cell lines to target early stages of cytosolic invasion, before viruses accomplish gene transcription and replication. As a result, ADX may be a useful model system in which to study ubiquitin-stimulated xenophagy and compare it between pathogen types. We have also established an automated imaging-based approach that allows for real-time, high-throughput quantification of bacterial hyper-proliferation and restriction inside live cells. Our method has advantages over traditional CFU assays and previously reported florescence-based approaches, which sacrifice temporal resolution, throughput, or automation.^91–94^ This may facilitate a more integrative understanding of pan-specific xenophagy.

Importantly, our data explain how TRIM21 can degrade large and highly complex substrates. The need to intercept and destroy phylogenetically and structurally diverse pathogens may have driven the evolution of TRIM21’s very broad substrate versatility. Its surprising and unique adaptability for TPD may derive from this built-in mechanistic flexibility. Unlike Cullin RING ligases, which must fit substrates into their E3 ligase active sites, TRIM21 molecules assemble around their targets and are therefore able to accommodate a wider range of substrate architectures. Selective autophagy is itself a highly versatile degradative system, and the ability of TRIM21 to induce autophagy of suitable substrates explains its capability as a broad-acting, pan-specific inducer of degradation. Considerable effort has recently been focused on the development of TPD technologies that provoke lysosomal degradation, which is important for targeting neosubstrates the proteasome cannot clear.^95–98^ Following the findings presented here, TRIM21 may provide a useful platform to trigger selective autophagy of neosubstrates by using small molecules.

## Limitations of the study

- Guide RNAs that caused growth defects tended to drop out completely from the whole-genome screen, and this limited its ability to report on essential pathways. Although we attempted to mitigate this with a focused screen using transient knockdown, it remains possible that we were unable to detect some effectors of TRIM21-mediated restriction for this reason.
- It is unclear what role UBE2N is playing in the initiation of ADX. It may be that K63 ubiquitin chains are directly modifying the substrate or that UBE2N has a regulatory role in autophagy initiation upstream of TRIM21 engagement. This will need to be disentangled in future work.
- Many pathogens have evolved mechanisms to evade xenophagy, but the study of viral/bacterial modulators of ADX are outside the scope of this study.

## Star★Methods

Detailed methods are provided in the online version of this paper and include the following:

- KEY RESOURCES TABLE
- EXPERIMENTAL MODEL AND STUDY PARTICIPANT DETAILS
  ○ Cell lines
  ○ Model pathogens
  ○ Mice
- METHOD DETAILS
  ○ Plasmids and cloning
  ○ Genetic depletions
  ○ Genetic complementation
  ○ Cell lysate preparation and immunoblotting
  ○ Retroviral production and transduction
  ○ Adenovirus infections
  ○ Proteasome activity assay
  ○ Antibody labelling
  ○ Antibody electroporations
  ○ Immunofluorescence
  ○ Confocal Microscopy
  ○ Structured Illumination Microscopy
  ○ Cell library and genome-wide screen
  ○ Library extraction, sequencing, and hit-calling
  ○ *Salmonella* cell infections
  ○ Salmonella immune sera preparation
  ○ Passive transfer experiment
  ○ Enumeration of bacterial organ load by colony forming unit assay
  ○ Salmonella burden in spleen and liver quantification by Q-PCR
- QUANTIFICATION AND STATISTICAL ANALYSIS

## Star★Methods

### Key Resources Table

**Table:** 

REAGENT or RESOURCE	SOURCE	IDENTIFIER
Antibodies		
9C12^WT/H433A^	GenScript or Foss et al.^92^	RRID: AB_3111592
anti-adenovirus	Merck	AB1056; RRID: AB_11212049
anti-mouse HRP	Agilent	P0260; RRID: AB_2636929
anti-rabbit HRP	Invitrogen	31462
*anti-Salmonella*	Abcam	ab35156; RRID: AB_777811
anti-lipopolysaccaride	Abcam	ab8274; RRID: AB_306423
ATG10	Abcam	ab124711; RRID: AB_10974774
ATG101	Abcam	ab229235
COXIV	LI-COR	926-42214; RRID: AB_2783000
FK2 (Ubiquitin)	Enzo Life Sciences	BML-PW8810; RRID: AB_10541840
GFP	Novus	NB600-303; RRID: AB_10001300
goat anti-human Fab AF-488	Cohesion Biosciences	CSA3203
LAMP1	Abcam	ab25630; RRID: AB_470708
LC3 (western blot)	Cell Signaling Technology	12741; RRID: AB_2617131
LC3 (SIM, Ad5)	Abclonal	A19665; RRID: AB_2862723
LC3 (SIM, *Salmonella)*	Cell Signaling Technology	83506; RRID: AB_2800018
NBR1	Invitrogen	PA5-30085; RRID: AB_2547559
NDP52	Abcam	ab68588; RRID: AB_1640255
OPTN	Invitrogen	711879; RRID: AB_2723433
p62 (rabbit)	Invitrogen	PA5-20839; RRID: AB_11157045
p62 [2C11]	Abcam	ab56416; RRID: AB_945626
T6BP	Sigma	HPA24432
TRIM21	Santa Cruz	cs25351
UBE2N	Invitrogen	PA5-17010; RRID: AB_10988857
β-actin HRP	Santa Cruz	sc47778; RRID: AB_626632
Bacterial and virus strains		
Human adenovirus serotype 5 ΔE1ΔE3 (CMV_EGFP)	Viraquest LLC	34760
Human adenovirus serotype 5 ΔE1ΔE3 (CMV_mCherry)	Viraquest LLC	34760
*Salmonella enterica* Typhimurium strain SL3261	Felix Randow	n/a
*Salmonella enterica* Typhimurium strain SL1344	Felix Randow	n/a
Chemicals, peptides, and recombinant proteins		
8-well IbiTreat	Ibidi	80806
Agarose Hi-Strength LMP	BioGene Ltd	300-800
AlexaFluor-647 N-hydroxysuccinamide ester	Invitrogen	37573
Amersham ECL reagent	Cytiva	RPN3004
Amicon Ultra 0.5 Centrifugal filter unit (100K)	Millipore	UFC510024
Autophinib	Selleck Chemical	S8596
Benzonase nuclease	Merck Millipore	E1014-5KU
BlasticidinS	Sigma-Aldrich	SBR00022
BugBuster protein extraction reagent	Merck	70584
Carfilzomib	Selleck Chemical	S2853
Cholera toxin	Sigma-Aldrich	C-8052
Complete Protease Inhibitor Cocktail	Roche	4693116001
Dextran-coated charcoal	Sigma-Aldrich	C6241
Dimethyl sulfoxide (DMSO)	Sigma-Aldrich	D8418
Dimethylformamide	Fisher Scientific	D/3840/08
Dithiothretol (DTT)	Thermo Fisher	R0861
DMEM/F12	Gibco	10565018
Doxycycline Hyclate	Sigma-Aldrich	D5207
Dulbecco’s Modified Eagle’s Medium (DMEM)	Gibco	11965092
Endothelial Cell Growth Media	R&D Biosystems	CCM027
Foetal Bovine Serum	Gibco	16000044
Fugene 6	Promega	E2691
Gentamycin	Amresco	E737
Gibson Assembly master mix	New England Biolabs	E2611L
HiScribe T7 ARCA mRNA kit	New England Biolabs	E2065S
Hoechst 33342	Abcam	ab228551
Horse Serum	Gibco	16050122
Human EGF recombinant protein	Peprotech	AF-100-15
Human Insulin recombinant protein	Sigma-Aldrich	I-1882
Hydrocortisone	Sigma-Aldrich	H0888-1G
iBlot transfer stack, nitrocellulose	Invitrogen	IB301001
Iscove’s Modified Dulbecco’s Media (IMDM)	Gibco	12440053
Ionic Detergent Compatibility Reagent for 660nm Reagent	Thermo Fisher	22663
KAPA SYBR Fast ROX low reagent master mix	Roche	KR0805
McCoy’s 5A	Gibco	16600082
MG-132	Sigma-Aldrich	M8699
MgCl^2^ (for PCR)	NEB	B9021S
MluI-HF	New England Biolabs	R3198S
Mowiol 4-88	Merck	81381
MRT68921 dihydrocholoride	Tocris Bioscience	5780
NanoGlo luciferase reagent	Promega	N1110
NEBuilder HiFi DNA assembly master mix	New England Biolabs	E2621L
Neon transfection kit	Thermo Fisher	MPK10096
NextSeq P2 reagent kit (200 cycles)	Illumina	20046812
NextSeq PhiX Control	Illumina	FC-110-3002
Non-fat dry milk	Marvel	N/A
NuPAGE 4-12% Bid-Tris mini protein gels	Invitrogen	NP0321
NuPAGE LDS Sample buffer	Invitrogen	NP0007
Optimem	Gibco	31985062
Paraformaldehyde, 16% solution	Thermo Fisher	11481745
Pierce 660nm Protein Reagent	Thermo Fisher	22660
Platinum SuperFi II PCR master mix	Thermo Fisher	12368010
Puromycin	Gibco	A1113803
Purple Gel Loading Dye (6X)	New England Biolabs	B7024S
QIAprep Spin Miniprep Kit	Qiagen	27104
QIAquick Gel Extraction Kit	Qiagen	28704
QuickChange Lightning	Agilent	210513
RIPA Buffer (10X)	Cell Signaling	9806
Sal1-HF	New England Biolabs	R3138S
Saponin	Sigma-Aldrich	47036-50G-F
sodium bicarbonate	Sigma-Aldrich	S5761
SYBR Safe DNA Gel Stain	Thermo Fisher	S33102
Syringe Filter 0.45 PVDF Membrane	Elkay Laboratory Products	E25-PV45
TAK-243	Selleck Chemical	S8341
TaqMan Fast Universal PCR Master Mix	Applied Biosystems	4352042
Triton X-100	Sigma-Aldrich	X100
Tween 20	Sigma-Aldrich	P1379-100ML
Viafect	Promega	E4981
Critical commercial assays		
Collibri Library quantification kit	Invitrogen	A38524100
DNeasy Blood and Tissue kit	Qiagen	69504
Proteasome-Glo Chymotrypsin-Like	Promega	G8660
Deposited data		
Sequencing data, unedited blots, imaging data	Mendeley	10.17632/cdgx63gd5y.1
Experimental models: Cell lines		
HEK293T	Ravi Gupta	N/A
HeLa WT (inhibitor panel)	Greg Towers	N/A
HeLa WT (5KO control)	Richard Youle	N/A
HeLa 5KO	Richard Youle	N/A
HUVEC	Felix Randow	N/A
MEF WT	Heo et al.^41^	N/A
MEF TRIM21^-/-^	Heo et al.^41^	N/A
MEF RNF213^-/-^	Felix Randow	N/A
MEF TRIM21^-/-^ RNF213^-/-^	This study	N/A
MCF10A	Buzz Baum	N/A
A549	ATCC	CCL-185
HT-1080	ATCC	CCL-121
RPE1	ATCC	CRL-4000
U2OS	ATCC	HTB-96
HAP1	Horizon Bioscience	C631
HEK293T-Cas9	This study	N/A
HEK293T-Cas9 sgRNA *TRIM21*	This study	N/A
HEK293T-Cas9 sgRNA *UBE2N*	This study	N/A
HEK293T-Cas9 sgRNA *ATG10*	This study	N/A
HEK293T-Cas9 sgRNA *ATG101*	This study	N/A
HEK293T-Cas9 sgRNA *UBE2N*	This study	N/A
HEK293T TRIM21 KO	Kiss et al.^50^	N/A
HEK293T TRIM21 KO pTMPP TRIM21	Zeng et al.^2^	N/A
HEK293T TRIM21 KO pTMPP msTRIM21	Zeng et al.^2^	N/A
HEK293T TRIM21 KO-Cas9	This study	N/A
HEK293T TRIM21 KO-Cas9 sgRNA *UBE2N*	This study	N/A
HEK293T TRIM21 KO-Cas9 sgRNA *ATG10*	This study	N/A
HEK293T TRIM21 KO-Cas9 sgRNA *ATG101*	This study	N/A
HEK293T TRIM21 KO FusionRed^MQV^-TRIM21	This study	N/A
HEK293T TRIM21 KO FusionRed^MQV^-TRIM21^I18R/M73E^	This study	N/A
HeLa 5KO + TetOne_p62-FLAG	This study	N/A
HeLa 5KO + TetOne_OPTN-EGFP	This study	N/A
HeLa 5KO + TetOne_NBR1-FLAG	This study	N/A
HeLa 5KO + TetOne_NDP52-FLAG	This study	N/A
HeLa 5KO + TetOne_TAX1BP1-FLAG	This study	N/A
HeLa 5KO + TetOne_EGFP	This study	N/A
Experimental models: Organisms/strains		
*Mus musculus* C57BL/6J	Jackson Labs	010724
*Mus musculus* C57BL/6J *TRIM21^-/-^*	Jackson Labs	010724
Oligonucleotides		
DNA oligonucleotides are listed in Table S3	This study	N/A
Recombinant DNA		
DNA plasmid sequences are listed in Table S3	This study	N/A
Software and algorithms		
R version 4.2.2	The R Foundation	https://www.r-project.org
‘ggplot2’	Hadley Wickham	https://ggplot2.tidyverse.org
‘lemon’	Stephen McKinnon Edwards	https://github.com/stefanedwards/lemon
CRISPick	Sanson et al.^99^	https://portals.broadinstitute.org/gppx/crispick/public
CHOPCHOP	Labun et al.^100^	https://chopchop.cbu.uib.no/
ImageJ (Fiji distribution) 2.1.0	Schneider et al.^101^	https://fiji.sc
Graphpad Prism 10.0	Dotmatics	https://www.graphpad.com
Original code	Mendeley	https://doi.org/10.17632/cdgx63gd5y.1
Other		
Brunello Lentiviral sgRNA library	Addgene	73178-LV

### Experimental Model and Study Participant Details

#### Cell lines

HEK293T cells were a gift from Ravi Gupta. HEK293T TRIM21 KO cells were previously reported.^58^ HeLa cells used in the inhibitor panel experiment were a gift from Greg Towers. HeLa WT and 5KO (KO *SQSTM1, NBR1, CALCOCO2, TAX1BP1, OPTN*) were a gift from Richard Youle.^61^ HUVEC and MEF RNF213^-/-^ cells were a gift from Felix Randow. MCF10A cells were a gift from Buzz Baum. A549, HT-1080, RPE1, and U2OS were purchased from ATCC. HAP1 cells were purchased from Horizon Bioscience. HEK293T-Cas9 were made by lentiviral transduction of pLentiCas9-Blast (Addgene, 52962) into HEK293T, followed by antibiotic selection.

HEK293T, HeLa, HAP1, and HT-1080 cells were cultured in DMEM (Gibco, 11965092) supplemented with 10% heat-inactivated foetal bovine serum (FBS; Gibco, 16000044). MEFs were cultured in IMDM (Gibco, 12440053) supplemented with 10% heat-inactivated FBS. U2OS cells were grown in McCoy’s 5A (Gibco, 16600082) supplemented with 10% FBS. RPE1 and A549 cells were grown in DMEM/F12 (Gibco, 10565018) supplemented with 10% FBS. HUVEC cells were grown in Endothelial Cell Growth Media (R&D Bio-systems, CCM027). Human breast MCF10A cells were grown in DMEM/F12 supplemented with 5% charcoal-stripped horse serum (Gibco, 16050122), 20 ng/mL epithelial growth factor (Peprotech, AF-100-15), 0.5 mg/mL hydrocortisone (Sigma-Aldrich, H08881G), 100 ng/mL cholera toxin (Sigma-Aldrich, C-8052), and 10 μg/mL insulin (Sigma-Aldrich, I-1882). All cell media was supplemented with 80 units/mL penicillin/streptomycin (Gibco, 15140122). Cells were incubated in 5% CO^2^ at 37 °C in a humid atmosphere. HEK293T, HeLa, HUVEC, MCF10A, RPE1, and U2OS cells are derived from human females. A549, HT-1080, and HAP1 cells are derived from human males. All cell lines tested negative for *Mycoplasma* at the beginning of the study.

Antibiotic selections were performed using 10 μg/mL blasticidin (Sigma-Aldrich, SBR00022) or 2.5 μg/mL puromycin (Gibco, A1113803). Tetracycline-inducible constructs were stimulated using 0.5 μg/mL doxycycline (Sigma-Aldrich, D5207). MG132 (Sigma-Aldrich, M8699) was used at 1 μM (2 μM in confocal microscopy experiments), carfilzomib (Selleck Chemical, S2853) and MRT68921 (Tocris Bioscience, 5780) at 1 μM, autophinib (Selleck Chemical, S8596) was used at 10 μM, and TAK-243 (Selleck Chemical, S8341) was used at 200 nM.

#### Model pathogens

Ad5 ΔE1ΔE3 CMV-EGFP or -mCherry was used for all infections (ViraQuest).

*S. enterica* serovar Typhimurium -mCherry, -mEGFP, and strains SL3261 and SL1344 were gifts from Felix Randow. For infection experiments, an overnight culture of *Salmonella* was sub-cultured 1:33 in lysogeny broth (LB) and grown for 3.5 hours 37 °C. This was used directly in cell experiments or centrifuged and resuspended in PBS for mouse infections. The viable count of the inoculum was confirmed by plating serial dilutions on LB agar.

#### Mice

All mice were maintained in the Medical Research Council ARES animal facility under pathogen-free conditions, at 19-23°C, 45-65% humidity, with a 12-h light-dark cycle. Animal experiments were undertaken with the approval of the Laboratory of Molecular Biology Animal Welfare and Ethical Review Body (AWERB) and the UK Home Office. At the beginning of the experiment, mice were matched for age, sex (equal numbers of each sex were used), and background strain. Mice were weighed twice daily and monitored for clinical signs of morbidity. At experimental endpoint, mice were euthanized by gradual exposure to CO_2_ followed by either cervical dislocation or exsanguination.

### Method Details

#### Plasmids and cloning

Plasmids pMD-OGD, m4p-EGFP-LC3B and m4p-EGFP-LC3C were gifts from Felix Randow. Plasmid pcRV1 was a gift from Stuart Neil, and pMD2.G was a gift from Didier Trono (Addgene, 12259). Plasmid pLentiCas9-Blast was a gift from Feng Zhang (Addgene, 52962), and pXPR_047 was a gift from David Root (Addgene, 107145). pTetOne was purchased from Takara Bio (634303).

Single guide RNAs in pXPR_047 (7.5 ng, Mlu1-Sal1) were constructed by Gibson HiFi assembly (New England Biolabs, E2621) of single-stranded DNA oligomers encoding crRNA and tracrRNA sequences with appropriate overlaps (2 pmol each, Integrated DNA Technologies). Assembly was performed in 10 μL volumes following the manufacturer-recommended protocol.

Autophagy adaptor sequences p62-FLAG, NDP52-FLAG, NBR1, OPTN-EGFP, and TAX1BP1 were provided as a gift in M6P plasmid backbones from Felix Randow. Along with *EGFP*, these were amplified by PCR and subcloned into pTetOne (Takara Bio, 634301).

Human codon-optimized FusionRed^MQV^ was ordered as a gene block with a C-terminal linker^56^ (Integrated DNA Technologies), and TRIM21 cDNA was amplified by PCR using sequences generated previously.^3^ FR-TRIM21 was constructed by Gibson assembly into an hPGK-driven pPMEZ backbone described previously.^102^ Mutations at I18R and M72E were introduced to FR-TRIM21 by site-directed mutagenesis (Agilent, 210513) using the primers: “18R *rev*”, “I18R *for*”, “M72R *rev*”, and “M72R *for*” (see Table S3). The tandem-fluorescent anti-Fab nanobody sequence^67^ was ordered as a gene block and cloned into pPMEZ.

After assembly, all plasmids were transformed into chemically-competent *E. coli* XL1-Blue, then plasmids were purified by mini-prep (Qiagen, 27104) and verified by sequencing. All DNA sequences made in this study can be found in Table S3.

#### Genetic depletions

TRIM21 KO HEK293T and MEF TRIM21^-/-^ were described previously.^49,58^ MEF RNF213^-/-^ were a gift from Felix Randow. MEF RNF213^-/-^ TRIM21^-/-^ double knockouts were generated by transfecting TRIM21 sgRNA/Cas9 ribonucleoprotein (IDT) into MEF RNF213^-/-^ cells and single-cell sorting clones into a 96-well plate using a previously-described sgRNA sequence and protocol.^11^ HEK293T-Cas9 knockout cell lines were produced by transducing pXPR_047 lentiviruses harbouring target sgRNAs, then selecting using puromycin. For transient genetic depletions in the targeted counter-screen, we co-transfected 65 ng pLentiCas9-Blast and 7.5 ng pXPR_047 with appropriate sgRNA sequence into HEK293T cells using 380 nL Viafect (Promega, E4981) in a 96-well plate. These cells were selected with antibiotics and assayed 5 days following transfection. All sgRNAs were designed using CRISPick^99^ or CHOPCHOP^100^ webservers, and sequences are listed in Table S3.

#### Genetic complementation

Stable expression of TRIM21 at endogenous levels in HEK293T KO TRIM21 cells (Figures 2C and S3H) was accomplished using native human or mouse *TRIM21* promotor-driven lentiviruses, as described previously.^2^ HeLa 5KO cells were complemented by transduction with lentiviruses harbouring pTetOne-autophagy adaptor sequences and selected using puromycin. Expression was induced by 24-hour treatment with doxycycline before infection experiments or immunoblot. HEK293T KO TRIM21 or MEF^WT^ cells were complemented with FR-TRIM21^WT^ or FR-TRIM21^I18R/M72E^ by lentiviral transduction. We used FACS to select FusionRed-positive cells which expressed FR-TRIM21 at levels as near endogenous TRIM21.

#### Cell lysate preparation and immunoblotting

Clarified RIPA lysates were prepared in RIPA buffer (Cell Signaling, 9806) supplemented with COmplete protease inhibitor cocktail (Roche, 4693116001) and benzonase (Merck Millipore, E1014). Lysis of cell pellets was performed on ice with gentle vortexing for 15 minutes, then lysates were cleared by centrifugation. 660-nm protein assay (Thermo Fisher, 22660) was used for lysate protein normalization. Samples were prepared for SDS-PAGE in 1X NuPAGE LDS buffer (Invitrogen, NP0007) and 100 mM dithiothreitol (Thermo Fisher, R0861) incubated for 5 min at 98 °C before loading. Electrophoresis was performed at 190V across NuPAGE 4-12% Bis-Tris precast gels (Invitrogen, NP0321) and electroblotted onto nitrocellulose membranes using semi-dry iBlot transfer (Invitrogen). Blots were blocked using 5% non-fat dry milk in 0.1% Tween 20 (Sigma-Aldrich, P1379) for one hour. Primary antibodies were incubated overnight, and secondaries for 1 hour at 4 °C. Antibodies used for immunoblotting were: TRIM21 (Santa Cruz, cs25351), β-actin HRP (Santa Cruz, sc47778), UBE2W (Invitrogen, PA5-67547), UBE2N (Invitrogen, PA5-17010), EGFP (Novus, NB600-303), anti-adenovirus (Merck, AB1056), ATG101 (Abcam, ab229235), ATG10 (Abcam, ab124711), LC3 (Cell Signaling, 12741) T6BP (Sigma-Aldrich, HPA24432), NBR1 (Invitrogen, PA5-30085), p62 (Invitrogen, PA5-20839), NDP52 (Abcam, ab68588), Optineurin (Invitrogen, 711879), anti-mouse HRP (Agilent, P0260), and anti-rabbit HRP (Invitrogen, 31462).

#### Retroviral production and transduction

Pseudotyped lentiviruses or γ-retroviruses were produced by co-transfecting HEK293T cells with pCRV (lentiviral Gag-Pol) or pMD-OGD (γ-retroviral Gag-Pol), and pMD.2G (VSV-G Env) with desired transfer plasmids using FuGENE 6 (Promega, E2691). Three days following transfection, viruses were collected from supernatant, filtered through 0.45 μm polyvinylidene fluoride membranes, and stored at -70 °C. Except for CRISPR library production (see below), cells were transduced in 2.5 μg/mL polybrene at an MOI ∼0.1. Antibiotic selections were performed at least two days following transduction.

#### Adenovirus infections

Except as otherwise specified, cells were seeded in 96-well plates the evening before inoculation. Humanised 9C12^WT^ and 9C12^H433A^ were engineered as described previously^103^ and were a gift from Jan Terje Andersen or were custom-ordered from GenScript. 2.2×10^7^ transforming units (TU, based on manufacturer data) of Ad5 was incubated with 80 ng 9C12 (unless otherwise noted) in 100 μL of complete DMEM for 30-60 min at room temperature. Where annotated as a concentration, 80 μL of the indicated concentration was combined with 2.2×10^7^ Ad5 in 20 μL for 30-60 minutes. Complexed 9C12:Ad5 was further diluted 1:3 in complete DMEM, then 20 μL of the suspension was inoculated on adherent cells, for a total dose of 1.5×10^6^ TU per well. For readout of viral genome delivery to the nucleus, EGFP or mCherry fluorescence was detected sixteen to twenty-four hours after inoculation using live-cell imaging or flow cytometry. The dose of Ad5 was sufficient to achieve ∼40% fluorophore-positive cells in control wells at 16 hours post-infection. For imaging-based quantification, fluorescent cells were counted on-plate using an IncuCyte (Sartorius). For flow cytometry, cells were detached and fixed in 2% formaldehyde, then detected using a CytoFlex (Beckman Coulter). GFP fluorescence was gated using uninfected control cells, and at least 5000 cells were counted per condition. After quantification, infected cell counts were normalized by total cells and compared to non-neutralizing controls.

For the hypothesis-driven focussed screen, cells transiently depleted for selected genes were inoculated with Ad5 as above, except the diluted virus was complexed 1:1 (v/v) with 0.8 μg/mL 9C12. Infected cells were measured by IncuCyte 16 hours after inoculation for 3 independent replicates. Infection with each sgRNA was quantified and normalized to confluency and infection controls. Data were filtered to exclude sgRNAs that led to substantial growth defects (<30% confluence the day after plating). At least 2 sgRNAs per gene were pooled and compared to cells expressing non-targeting control sgRNAs to calculate an estimated fold-change of infection. For per-gene enrichment, *P* values were calculated by 1-way ANOVA and corrected for false discovery using Dunnett’s test. For groupwise enrichment in Figure S3A, nested 1-way ANOVA was performed and corrected using Dunnett’s Test. Genes lacking at least 3 data points were excluded from analysis. Results are plotted as fold-change in infection, relative to NTC cells.

For the panel of cells treated with inhibitors in Figure 2C, infection was performed as above, but cells were pre-treated with inhibitors for two hours before infection. For the cell panel experiment in Figure S3H, infection was synchronized by centrifugation at 800 rcf, 8 °C for 30 minutes. Following this, inhibitors were added, cells were temperature-shifted to 37 °C to synchronize infection, and media exchanged 1 hour later. For some cell lines with lower permissivity to Ad5, the inoculum concentration was scaled to achieve ∼40% positive cells in control wells.

For time-of-addition experiments with inhibitors in Figure 2D, cells were pre-treated with compounds for the indicated durations, then infection was synchronized by centrifugation at 800 rcf, 8 °C for 30 minutes. Following this, cells were temperature-shifted to 37°C and media exchanged 1 hour later. For the condition in which inhibitors were added 1 hour after infection, this was done at the time of the media change and left on cells for the remaining duration of infection.

For immunoblotting following infection (Figures S3E, S6E, and S6F), Ad5 cell entry was synchronized as above. Inhibitors were added at the time of temperature shift and removed 1 hour later. At the indicated timepoints, cells were trypsinised, washed twice in PBS, and lysed in Laemmli buffer containing protease inhibitors before gel electrophoresis.

For spinning-disk confocal microscopy experiments, cells were seeded the day prior to infection (with or without electroporation, see below) in 8-well IbiTreat dishes (Ibidi, 80806) coated with poly-L-lysine solution (Sigma-Aldrich, P4707). 5×10^6^ TU of Ad5 were incubated with 2 μg of AlexaFluor 647-labelled 9C12 in complete media at room temperature for 1 hour in the dark. Cells were then chilled, inoculated with AF-9C12:Ad5 complexes, and incubated on ice for 30 min to synchronize infection. Inoculate was then removed and replaced with media at 37°C to initiate viral entry. Cells were imaged live or wells were fixed at various time points using 4% formaldehyde in PBS before immunostaining. For super resolution structured illumination microscopy, conditions were the same except cells were seeded on high-precision size 1.5 coverslips (Carl Zeiss) and fixation was performed with ice-cold methanol.

For split-luciferase assays, mRNA was in vitro transcribed from linearized pGEMHE plasmids containing sequences for SmBiT-TRIM21, LgBiT-TRIM21, or LgBiT-TUBEs using the HiScribe T7 ARCA kit (NEB) and following manufacturer instructions. HEK293T TRIM21 KO cells were electroporated with 0.25 μM of the indicated mRNAs (or PBS) the day before the experiment. Synchronized infections with 9C12^WT^-complexed adenovirus were performed as described above, and cells were lysed in Nano-Glo lytic luciferase reagent (Promega) 30 minutes after temperature shift. Luciferase activity was measured using a GloMax plate reader (Promega). Measurements were plotted as the fold-luminescence relative to the average luminescence of PBS-electroporated cells.

#### Proteasome activity assay

Chymotrypsin-like proteasome activity was measured using the live-cell luminescence based Proteasome-Glo kit (Promega), following manufacturer instructions. Inhibitors were added as indicated 10 minutes following addition of the luminescence substrate to the media.

#### Antibody labelling

9C12 and anti-*Salmonella* (Abcam, ab35156) antibodies were labelled with AlexaFluor-647 N-hydroxysuccinamide (NHS) ester (Invitrogen, 37573). 100 μg of the AF-NHS ester was dissolved in 100 μL of dimethylformamide (Fisher Scientific, D/3840/08) to make the dye. 100 μg of antibody was diluted in 50 μL of PBS and mixed with 25 μL freshly made 50 mM sodium bicarbonate (Sigma-Aldrich, S5761) and 8 μL of freshly prepared dye (1:10 antibody:dye molar ratio). The mixture was incubated at room temperature for 1 hour in the dark. Labelled antibodies were purified and concentrated to ∼ 2 mg/mL using Amicon Ultra 0.5 ml centrifugal filters with 100 kDa cut off (Millipore, UFC5100) and stored in small aliquots at -70°C.

#### Antibody electroporations

Anti-*Salmonella* (1 μg), 9C12 (0.5 μg), or anti-Fab (0.5 μg) antibodies were mixed with at least 200K cells in suspension, and electroporated using a Neon transfection system (Thermo Fisher) set to 2×20 millisecond pulses of 1400V. Cells were recovered in complete DMEM lacking antibiotics and plated on 8-well IbiTreat dishes for confocal microscopy the following day.

#### Immunofluorescence

To permeabilize fixed cells, samples were incubated with 0.1% Triton X-100 (Sigma-Aldrich, X100) or with 0.05% saponin for LAMP1 staining. After blocking with 5% FBS in PBS, wells were incubated sequentially with primary and secondary antibodies for 1 hour at room temperature. The FK2 antibody was incubated overnight at 4°C. Nuclei were stained with Hoechst 33342 (Abcam, ab228551).

For some experiments AlexaFluor-647 labelled antibodies or anti-human Fab AF-488 were electroporated into cells the day prior to infection. Antibodies used for IF experiments were: mouse p62 [2C11] (Abcam, ab56416); rabbit p62 (Invitrogen, PA5-20839); NDP52 (Abcam, ab68588); LAMP1 (Abcam, ab25630); polyubiquitin FK2 (Enzo Life Sciences, BML-PW8810); goat anti-human Fab AF-488 (Cohesion Biosciences, CSA3203); secondary antibodies were from Invitrogen.

#### Confocal Microscopy

All images were acquired on a Nikon W1 spinning disk inverted microscope with 100x/1.4NA oil objective and sCMOS camera. At least four images of each condition were taken and used for analysis. The proximity of puncta in several channels were evaluated using custom-made workflows in ImageJ^101^ and CellProfiler.^104^ Within each experiment, images were acquired with identical instrument settings and batch-processed together.

For per-image analysis of Ad5 infection experiments in ImageJ (Figures 2F, 2J, 2K, 3D–3I, and S4C), blob-like structures were detected in 3D using a custom difference of Gaussian approach at a pre-defined scale where local maxima above a threshold were selected in each channel. Sub-pixel localization was then achieved using block-coordinate Gaussian least square curve fitting. Proximity events were recorded for each localization when the centroids of two object markers were within 0.5 μm of each other. For percell analysis of Ad5 infection with cellular markers (Figures 3J and S4E), projections were generated in ImageJ and then cells, nuclei, and spots were segmented and assigned in CellProfiler using Cellpose.^105^ Omnipose^106^ was used to identify punctate markers, and statistical analysis was performed in R. For enumeration of fluorescent bacteria (Figures 4A, S5A, and S5C), Omnipose was used with: –pretrained_model bact_fluor_omni –mask_threshold 0.0 –flow_threshold 0.0 –max_size 200. Identified masks were then imported to CellProfiler and per-bacteria colocalization with markers was calculated using a custom pipeline. For analysis of FK2 fluorescence intensity of cytosolic bacteria, we measured the integrated FK2 intensity around bacteria which colocalized with electroporated antibodies and scored as FK2-positive. Before plotting, FK2 intensity values were averaged per-image and normalized to control conditions. For some fluorescence images with bacteria, per-channel intensity was plotted on a line profile using the ‘Plot Profile’ function in ImageJ and normalized to 1. The ImageJ and CellProfiler pipelines, R scripts, and Omnipose settings for all experiments are included in archived data deposits at BioStudies (accession S-BSST1877).

#### Structured Illumination Microscopy

HEK293T or MEF cells were grown on high-precision size 1.5 coverslips (Carl Zeiss), and infected with Ad5 or *S*. Typhimurium as described. Ammonium chloride treatment was performed overnight before infection. Following infection, cells were fixed in cold methanol and stained for conventional fluorescence microscopy. Coverslips were mounted with Mowiol mounting medium (Merck).

Microscopy was performed using an Elyra PS1 instrument (Carl Zeiss). Samples were examined on the microscope using a 63x 1.4NA plan-apo Carl Zeiss objective lens and Immersol 518F (23°C) immersion oil. Image acquisition was carried out using ZEN 2012 Elyra edition software in which data sets were collected with 5 grating phases, 5 rotations and sufficient z positions spaced 110 nm apart to form a 2mm deep volume of raw SR-SIM data. Optimal gratings were selected for each wavelength used. Structured Illumination post-processing was performed in ZEN using parameters determined by automated analysis of the datasets. Reconstructed images were then corrected for spherical and chromatic aberrations using channel alignment information which was created using a 3D array of multi-spectral beads previously imaged with the same instrument settings. Viruses and bacteria colocalizing with LC3 were counted by hand from at least 6 separate fields. For bacteria quantifications, LC3 coats were considered “full” if a distinct ring of LC3 could be observed around the bacteria. “Partial” coats were counted if any amount of LC3 colocalized with bacteria.

#### Cell library and genome-wide screen

Cas9 activity was verified by transducing low-titre of lentivirus encoding EGFP and sgRNA against GFP (pXPR_047) as previously described.^99^ Briefly, HEK293T WT or HEK293T-Cas9 cells were transduced with equal amounts of pXPR_047 lentivirus and selected using puromycin. Two weeks after selection, cells were detached, fixed in 2% PFA, then analysed for GFP fluorescence by flow cytometry on a CytoFlex flow cytometer. Negative control HEK293T cells were used to create a GFP+ gate. Efficiency of Cas9 was estimated as the percent loss of fluorescence of Cas9+XPR cells compared to the WT+XPR control (∼85% from data in Figure S1A). Data were analysed using Floreada.io software.

The Brunello library in pLentiGuide-Puro was purchased as lentiviral particles (Addgene, 73178-LV). These were titred in HEK293T-Cas9 cells by inoculating the cells in 6-well plates and selecting in puromycin.^99^ A range of volumes of the lentivirus preparation was added to 293T-Cas9 cells seeded in 6 well plates. Two wells were inoculated per virus volume. The day after seeding, the inoculum was replaced with fresh media. Two days after infection, puromycin was added to one of the paired virus wells. Three days later, cell confluency was measured using an IncuCyte widefield microscope. A viral dose resulting in ∼30% transduction efficiency was then used to inoculate 133 million HEK293T-Cas9 cells for the cell library. Two days after transduction, cells were selected in puromycin. The library was cultured for 10 days with selection before placing in liquid nitrogen storage. At every passage, enough cells were carried over to maintain a library coverage of 500X per guide.

For every biological replicate, fresh samples of the cell library were recovered from nitrogen storage. These were cultured for 1 week, then enough of the cell library was inoculated with Ad5 ΔE1ΔE3 GFP to ensure 100X coverage per guide after the cell sort. For Ad5 control infections, 21.9 M cells were inoculated per replicate; for Ad5 complexed with 9C12^H433A^ 43.8 million cells were inoculated; for Ad5 complexed with 9C12^WT^ 131.4 million cells were inoculated. Before each inoculation, Ad5 was incubated with the appropriate 9C12 mutant (concentration in incubation volume: 1 μg/mL) or PBS for 1 hour at room temperature. 16-20 hours after inoculation, live cells were sorted via FACS into GFP+ and GFP- gates. We targeted an MOI ∼0.6 for Ad5 control infections; this meant the effective MOI was 0.12 for 9C12^WT^ and 0.55 for 9C12^H433A^ infections. We sorted 8 million cells into the GFP+ gate for each biological replicate of each condition. For library collection, 21.9 million cells were inoculated with control Ad5, and 8 million of these were collected 16 hours later, without sorting. After sorting/collection, cells were pelleted by gentle centrifugation and snap frozen in liquid nitrogen. Three independent biological replicates were performed for all experimental groups, and 2 library samples were collected.

#### Library extraction, sequencing, and hit-calling

Two-step genome amplification was used to extract incorporated guide sequences from sorted cells for deep sequencing. Genomic DNA was extracted using DNeasy Blood and Tissue extraction (Qiagen, 69504) following manufacturer instructions, except 2.5 million cells were loaded per column. For the first PCR, all extracted genomic DNA was amplified using primers flanking the sgRNA sequence. Amplification of genomic sgRNA sequences was performed in 5 μg fractions/50 μL reaction, using SuperFi II DNA polymerase master mix (Thermo, 12368010) spiked with 1 mM MgCl_2_. The thermocycler was set for cycles of 10s at 95 °C, 10s at 60 °C, and 30s at 72 °C. After 18 cycles, a final 5 minute 72 °C incubation was performed. Next, PCR products were pooled by replicate and quantified using KAPA SYBR Fast ROX low reagent master mix (Roche, KK4973) using manufacturer-recommended settings: 40 cycles of 95 °C for 30s, 53 °C for 30s, 72 °C for 30s. The CT values from three technical replicates were averaged, and the lowest average CT value from any of the replicate samples was used to determine the number of cycles for the second PCR. In the second PCR, condition- and replicate- barcoded Illumina adaptors were appended to the amplified guide sequences. Forward P5 primers were a pool with stagger sequences inserted between the multiplexing read 1 sequencing primer site and hU6 promoter sequence to increase diversity on the flow cell. Reverse P7 primers contained barcodes for sample-and-replicate multiplexing. SuperFi II master mix was used with cycling settings as above. PCR products were then loaded onto a 2% low-melting point agarose gel, and purified bands were cut out before gel melting at 40 °C and purification (Qiagen, 28704). Amplified DNA was next normalized between replicates based on qPCR using the Collibri library quantification kit (Invitrogen, A38524100) following manufacturer instructions. All primer and adaptor sequences used for the genome-wide screen can be found in Table S3.

Normalized, barcoded DNA was pooled and spiked with 15% PhiX high-diversity control library (Illumina, FC-110-3001). Sequencing was performed using an Illumina NextSeq with P2 200 sequencing kit (Illumina, 20046812) following manufacturer instructions. Sequences were aligned and guides counted as described previously.^107^ Demultiplexing was performed using demuxFQ. Flanking sequences were removed using cutadapt-1.4.1 (analysis parameters: -e 0.2 –minimum-length 20 –discard-untrimmed). The flanking sequences were: GACGAAACACCG and GTTTTAGA for the 5’ and 3’ ends, respectively. The 20bp reads were then matched to the Brunello reference library and counted (cutadapt parameters: -f -v 0 -m 1 –norc -a –best –strata - -un). Guides with less than 20 and more than 1500 counts in the unsorted library were filtered from all groups and each biological repeat was analysed independently using stat.wilcox in caRpools v0.83. Raw sgRNA counts per replicate can be found in Table S2. The counts were then normalized to the median of the population within each biological replicate. For each comparison, four sgRNAs targeting a single gene were pooled and enrichment scores calculated for the pool. The p-values were calculated using a two-sided Mann-Whitney-U test. Then, data from each replicate were combined by calculating the mean enrichment score and p-values were combined using Fisher’s method and adjusted for false-discovery using the Benjamini-Hochberg method. Hits were considered as those genes with caRpools enrichment scores greater than 0.3 and adjusted p<0.01. Enrichments scores for all genes across all comparisons can be found in Table S1.

For genetic pathway enrichment analysis, genes from each comparison were ranked by their caRpools enrichment score, and the top 30 from each comparison were pooled. Duplicates were removed and the network was queried against the human genome background using STRING^108^ with default settings. Nodes were coloured based on functional enrichment. For the functional pathway analysis in Figure S1D, we queried the same enrichment list against GO: Cellular Component using ShinyGO 0.82. The minimum pathway size was 10, and FDR was set to 0.05. Results were manually curated to remove redundant entries. The Euler diagram in Figure 1F was made in R using the ‘eulerr’ package.

#### *Salmonella* cell infections

For tracking bacterial growth rate in infected MEFs, cells were plated in antibiotic-free IMDM on 96-well plates the day before infection. The day of infections, serial dilutions of anti-*Salmonella* (Abcam, ab35156) or anti-GFP (Novus, NB600-303) were made. 24 μL of *S*. Typhimurium-mCherry subculture was incubated with 120 μL of each antibody concentration for 10 minutes at room temperature. These were then diluted 1:5 and 20 μL was inoculated per well. Cells were allowed to infect for 30 minutes at 37 °C, then were washed several times and the media was changed to IMDM containing 100 μg/mL gentamycin (Amresco, E737) to disable any remaining extracellular bacteria. Infected cells were immediately moved to an IncuCyte for live-cell imaging. For measurement of initial multiplicity of infection, individual fluorescent bacterial spots were counted and normalized to MEF confluency. For quantification of growth rate, we used IncuCyte software to segment specifically for hyperproliferating fluorescent bacteria, which mark the cytosolic fraction. We then calculated a per-cell rate-of-change in fluorescence intensity over the indicated timespans.

For confocal imaging of *Salmonella* infections, MEFs were plated on 8-well IbiTreat dishes the day before infections. For each well, 40 μL of *S*. Typhimurium-EGFP subculture was diluted with 20 μL of 15 μg/mL anti-*Salmonella* or control antibody, incubated for 10 minutes at room temperature, then inoculated on cells. For some experiments, fluorescent antibody was electroporated into cells the day before infection, and cells were infected directly with uncoated bacteria. Following gentamycin media change 30 minutes after inoculation, wells were fixed at desired timepoints in 4% formaldehyde. Infections were identical for structured illumination microscopy, except MEFs were plated on high-precision size 1.5 coverslips (Carl Zeiss) and fixed in ice-cold methanol.

For immunoblotting, MEFs were plated in 6-well format and infected with 133 μL *Salmonella* subculture which had been incubated with 66 μL of 15 μg/mL anti-*Salmonella* antibody for 10 minutes at room temperature. Cells were changed into gentamycin-containing media as above, then lysed using BugBuster protein extraction reagent (Merck) containing protease inhibitors.

For split-luciferase experiments, His-lipoyl-SmBit-TRIM21 and His-lipoyl-LgBit-TRIM21 proteins were recombinantly expressed in E. coli (C41) in 2XTY media (supplemented with 0.5% glucose, 2 mM MgSO4 and appropriate antibiotics) at 37 °C for 2–3 h (OD600 around 0.6-1) after which they were induced with 1 mM isopropylthio-β-galactoside (IPTG) and incubated at 18 °C overnight. Cells were pelleted with a Sorvall SLC-6000 compatible centrifuge at 4500 × g for 25 min. The pellet was resuspended in lysis buffer (50 mM Tris pH 8, 1 M NaCl, 10% v/v BugBuster (Novagen), 10 mM imidazole, 2 mM DTT and 1 × cOmplete protease inhibitors (Roche) and sonicated for 15 min total time (10 s on/20 s off) at 70% amplitude. The soluble fraction was recovered by centrifugation at 40 000 × g in a JLA25.50 rotor. The clarified lysate was applied to a gravity flow column prepared with 5–10 ml of NiNTA agarose (Qiagen) equilibrated with Buffer B (300 mM NaCl, 50 mM Tris pH 8, 10 mM imidazole and 1 mM DTT). The bound fraction was washed in Buffer B (∼ 1–20 bed volumes) and eluted with Buffer E (300 mM NaCl, 50 mM Tris pH 8, 400 mM imidazole and 1 mM DTT). Fractions of about 2 mL were collected for about 15–30 mL of eluate. Fractions containing the protein were pooled, filtered and separated by Size-Exclusion Chromatography (SEC) using HiLoad 26/600 Superdex 75/200 pg columns (Cytiva) in 150 mM NaCl, 50 mM Tris pH 8 and 1 mM DTT. 1 pmol of each purified dimeric SmBit-TRIM21 and LgBit-TRIM21 protein were mixed with 2 pmol anti-lipopolysaccaride antibody (Abcam) and 25 μL Luria broth or *S*. Typhimurium subculture (O.D. ∼ 0.2) and 1:100 nanoluciferase reagent (Promega) in a total volume of 100 μL. Glow-type luciferase signal was measured on a GloMax Discover plate reader (Promega) and normalized to condition lacking luciferase protein.

#### Salmonella immune sera preparation

12.5-week-old WT C57BL/6J or TRIM21^-/-^ (Jackson Laboratory strain 010724) mice (6 in each group) were inoculated with 1×10^5^ colony forming units (CFU) of SL3261 via the tail vein. Mice were boosted twice: at day 21 post-infection with 4×10^6^ CFU of SL3261 intravenously, and at day 36 with 1×10^4^ CFU of SL1344 by intraperitoneal (IP) injection. Mice were culled and exsanguinated at day 45, and cell-free sera was prepared from collected blood. Sera was heat-inactivated and titred by ELISA against *S*. Typhimurium coated on a 96-well plate. Sera from the top 9 responders was pooled and diluted 1:50 for passive transfer.

#### Passive transfer experiment

10-week-old WT C57BL/6J or TRIM21^-/-^ mice (6 per group) were administered 100 μL diluted *S*. Typhimurium antisera or PBS by IP injection 2 days before infection. Mice were infected with 30 CFU SL1344 by IP injection. On days 1 and 3 post injection, they received 100 μL of diluted sera or PBS boost. Six days after infection, mice were euthanized and liver and spleen were collected. One half of each organ was snap frozen in liquid nitrogen and the other was placed on ice. During the experiment, two animals (one WT without serum and one TRIM21^-/-^ without serum group) became moribund and were euthanized before experimental endpoint. These were excluded from statistical analyses.

#### Enumeration of bacterial organ load by colony forming unit assay

Fresh collected tissues were weighed and thoroughly homogenized in cold PBS. Disrupted tissues were combined with Triton X-100 (Sigma-Aldrich, X100) to a final concentration of 0.1% and incubated on ice for 10 minutes. Serial 10-fold dilutions were made in PBS, and these were plated on LB agar plates. After overnight incubation at 37°C, plates with discrete colonies were counted and the bacterial load per milligram of tissue was calculated.

#### Salmonella burden in spleen and liver quantification by Q-PCR

Genomic DNA of frozen tissue samples was extracted with a DNeasy Blood and Tissue kit (Qiagen, 69504) according to the manufacturer’s protocol. Each qPCR reaction contained 5 μL TaqMan Fast Universal PCR Master Mix (Applied Biosystems, 4352042), 0.9 μM primers, 0.25 μM probe, 3 μg gDNA and water to 10 μL. A StepOnePlus Real-Time PCR System (Applied Biosystems) was used for thermocycling. The protocol was: 2 min at 50°C, 20 sec at 95°C, then 40 cycles of 15 sec at 95°C and 20 sec at 60°C. To produce the standard curve, genomic DNA was extracted from an overnight culture of *S*. Typhimurium SL1344 and serially diluted from 10^8^ to 1 genome-equivalent copies per μL. Primers and probe used for the detection and quantification of *S*. Typhimurium were as previously described^109^: Sal *for* (GCGCACCTCAACATCTTTC); Sal *rev* (CGGTCAAATAACCCACGTTCA); *probe* (FAMAATCATCGTCGACATGC-MGB/NFQ).

### Quantification and Statistical Analysis

Custom plots were coded in R using ‘ggplot2’ and ‘lemon’. Other data representation was performed using GraphPad Prism v10. Statistical analysis was done in R or GraphPad Prism. Sample sizes and statistical comparisons are indicated in the figure legends and methods. Throughout the study, experimenters were not blinded to subject assignments. Unless indicated otherwise in the methods, statistical significance was considered as *p*<0.05. For representation of statistical significance, where appropriate, asterisks are used to indicate *p*: <0.05 (*), <0.01 (**), <0.001 (***), <0.0001 (****). Figures were assembled in Adobe Illustrator 2024. Graphics were made using BioRender and Illustrator.

## Supplementary Material

Supplemental information can be found online at https://doi.org/10.1016/j.molcel.2026.04.031.

Figs S1 - S8

Table S1

Table S2

Table S3

## In brief

Rhinesmith et al. describe an intracellular antimicrobial mechanism called “antibody-directed xenophagy” (ADX). In ADX, the E3 ubiquitin ligase TRIM21 prevents infection by intercepting antibody-coated viruses/bacteria in the cytosol and orchestrating their destruction via selective autophagy. The authors hypothesize that ADX may be co-opted for targeted protein degradation.

## Highlights

- Antibodies direct viruses and bacteria for post-infection xenophagy
- TRIM21 regulates ADX via its ubiquitination activity
- ADX protects against pathogenic *Salmonella* infection *in vivo*

## Resource Availability

### Lead contact

Further information and requests for resources and reagents should be directed to and will be fulfilled by the lead contact, Leo James (lcj@mrclmb.ac.uk).

### Materials availability

All unique/stable reagents generated in this study are available from the lead contact with a completed materials transfer agreement.

### Data and code availability

- Next-generation sequencing data, unedited blots, and representative images have been deposited to Mendeley Data: https://www.doi.org/10.17632/cdgx63gd5y.1.
- Original code reported in this study has been deposited to Mendeley Data: https://www.doi.org/10.17632/cdgx63gd5y.1.
- Any additional information required to reanalyze the data reported in this paper is available from the lead contact upon request.
