# Supplementary material for: TRIM21 induces selective autophagy of viruses and bacteria
Source: Mol Cell. Author manuscript; Available in PMC 2026 Sep 8. (PMC7619466; doi:10.1016/j.molcel.2026.04.031)
Supplement: Supplementary file 1 [file EMS217847-supplement-Figs_S1___S8.pdf]

Molecular Cell, Volume *86*

Supplemental information

TRIM21 induces selective autophagy of viruses and bacteria

Tyler Rhinesmith, Anna Albecka, Marina Vaysburd, Claudia Puri, Jakub Luptak, Jerome Boulanger, Quynh-Mai Nguyen Le, Matthew J. Gratian, Kevin O'Connell, Lara Few, Mark Donaldson-Wing, Patrycja Kozik, David C. Rubinsztein, and Leo C. James

TRIM21 induces selective autophagy of viruses and bacteria

Tyler Rhinesmith,[1,*] Anna Albecka,[1] Marina Vaysburd,[1] Claudia Puri,[2,3] Jakub Luptak,[1,4] Jerome Boulanger,[1] Quynh-Mai Nguyen Le,[1] Matthew J. Gratian,[2] Kevin O'Connell,[1] Lara Few,[1] Mark Donaldson-Wing,[1] Patrycja Kozik,[1] David C. Rubinsztein,[2,3] and Leo C. James[1,5]**
[1]MRC Laboratory of Molecular Biology, Cambridge, UK
[2]Cambridge Institute for Medical Research, Cambridge, UK
[3]UK Dementia Research Institute, Cambridge, UK
[4]Present address: Protein Sciences, Structure, and Biophysics, Discovery Sciences, R&D, AstraZeneca, Cambridge, UK
[5]Lead contact
*Correspondence: rhinesmith@mrclmb.ac.uk
**Correspondence: lcj@mrclmb.ac.uk

Supplementary Information
Figures S1–S8

Figure S1. Validation of the genome-wide screen, related to Figure 1

(A) Endonuclease efficiency test in HEK293T-Cas9 cells used for the whole-genome screen. Loss of EGFP fluorescence was measured in cells transduced with the fluorescent protein and an sgRNA targeting *EGFP* compared to HEK293T cells lacking Cas9. Cutting efficiency was estimated at ~85% of transduced cells.

(B) Example gating of EGFP-positive HEK293T-Cas9 cells infected with Ad5 and analysed via FACS the following day.

(C) Fraction of cells which were gated EGFP+ in whole-genome screen replicates.

(D-E) 51 genes pooled from the top-30 enrichments in screen comparisons against $9C12^{WT}$, analysed for (D) GO: Molecular Function terms curated from ShinyGO v0.81 and (E) functional association network using STRING.

(F) Enrichment of sgRNAs targeting the indicated genes in cells infected with control Ad5 compared to the unsorted library.

(G) STRING functional association network for pooled genes among the top-30 enriched sgRNA targets from Ad5 alone and $Ad5{:}9C12^{H433A}$ compared to the unsorted library.

Fig S1

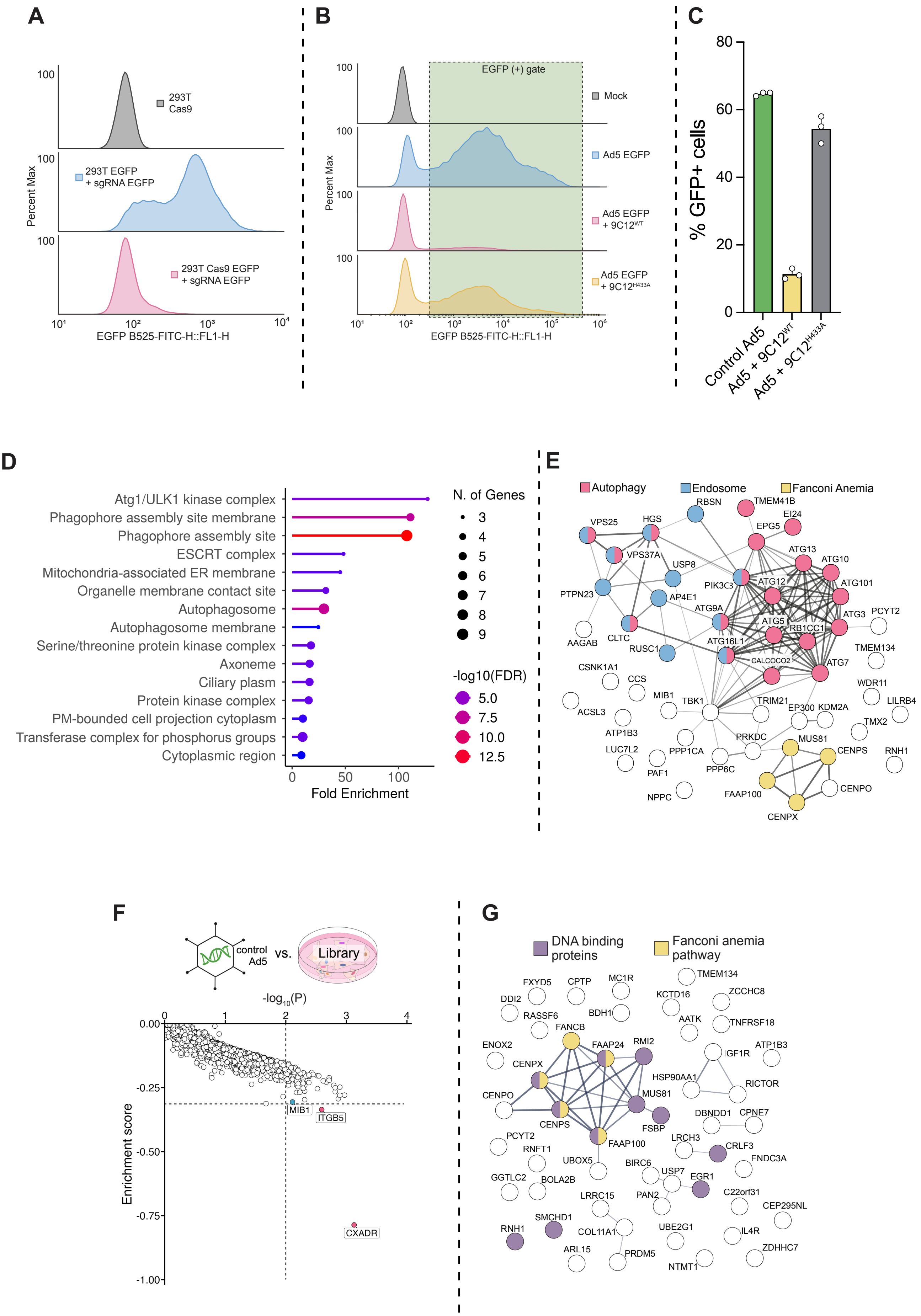

A
293T Cas9
293T EGFP + sgRNA EGFP
293T Cas9 EGFP + sgRNA EGFP
Percent Max
EGFP B525-FITC-H::FL1-H
B
EGFP (+) gate
Mock
Ad5 EGFP
Ad5 EGFP + 9C12WT
Ad5 EGFP + 9C12H433A
Percent Max
EGFP B525-FITC-H::FL1-H
C
% GFP+ cells
Control Ad5
Ad5 + 9C12WT
Ad5 + 9C12H433A
D
Atg1/ULK1 kinase complex
Phagophore assembly site membrane
Phagophore assembly site
ESCRT complex
Mitochondria-associated ER membrane
Organelle membrane contact site
Autophagosome
Autophagosome membrane
Serine/threonine protein kinase complex
Axoneme
Ciliary plasm
Protein kinase complex
PM-bounded cell projection cytoplasm
Transferase complex for phosphorus groups
Cytoplasmic region
Fold Enrichment
N. of Genes
-log10(FDR)
E
Autophagy
Endosome
Fanconi Anemia
F
control Ad5
vs.
Library
-log10(P)
Enrichment score
MIB1
ITGB5
CXADR
G
DNA binding proteins
Fanconi anemia pathway

Figure S2. Autophagy pathway components involved in TRIM21-mediated restriction of Ad5, related to Figure 1

(A) Illustration of the selective autophagy pathway, with hits from the whole-genome screen annotated in pink.

(B) $9C12^{WT}$:Ad5 infection in HEK293T-Cas9 cells transduced with indicated sgRNAs. Significance of effect was estimated from n=3 replicates against NTC cells by one-way ANOVA with Dunnett's test for multiple comparisons.

(C) Control Ad5-mCherry infection in cells depleted for the indicated gene products. No condition reached significance in n=3 replicates against the NTC condition in one-way ANOVA with Dunnett's test.

Fig S2

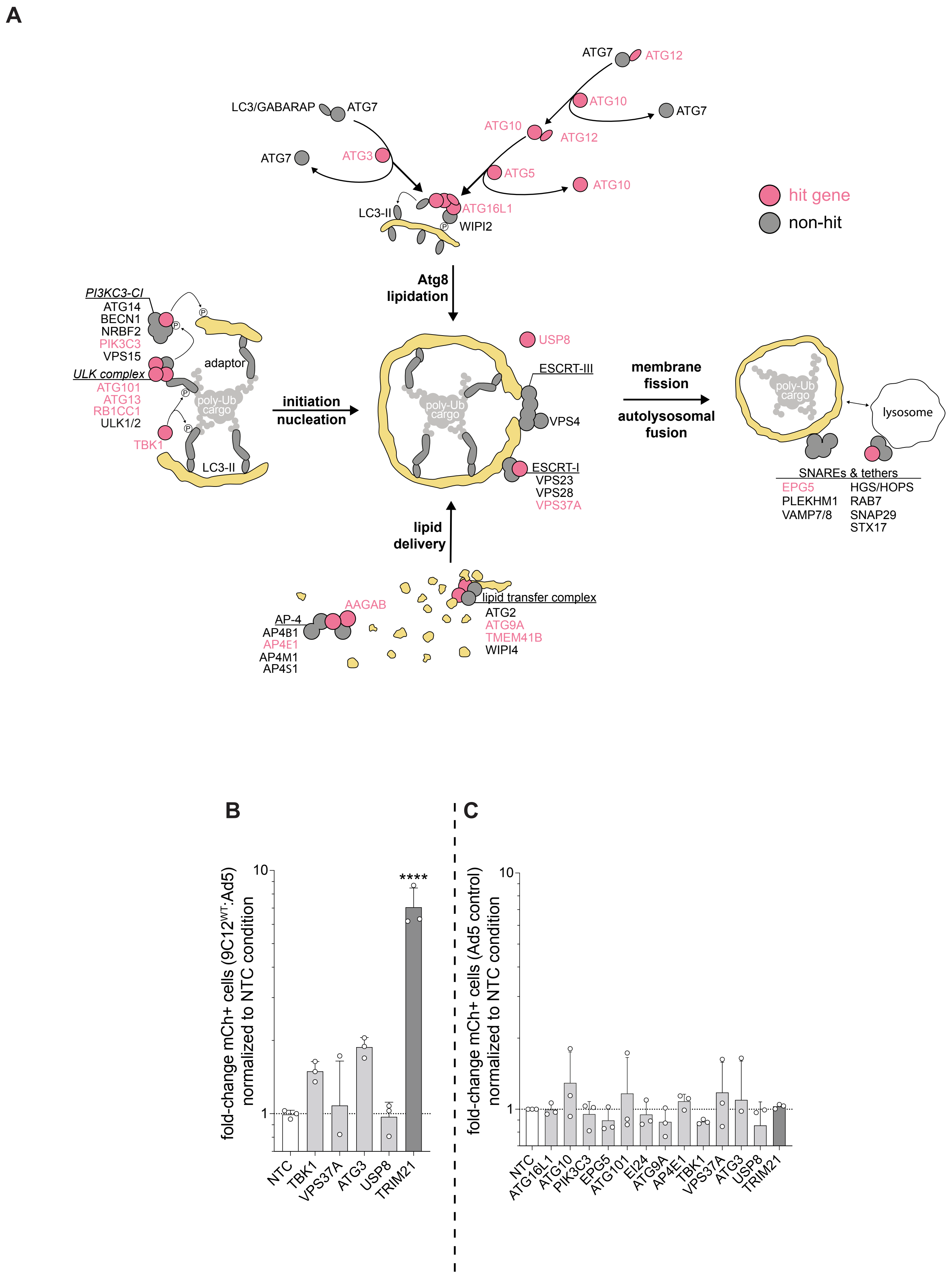
A
ATG7
ATG12
LC3/GABARAP
ATG7
ATG10
ATG7
ATG10
ATG12
ATG7
ATG3
ATG5
ATG10
LC3-II
ATG16L1
WIPI2
hit gene
non-hit
Atg8
lipidation
PI3KC3-CI
ATG14
BECN1
NRBF2
PIK3C3
VPS15
adaptor
ULK complex
ATG101
ATG13
RB1CC1
ULK1/2
TBK1
LC3-II
poly-Ub
cargo
initiation
nucleation
USP8
ESCRT-III
VPS4
ESCRT-I
VPS23
VPS28
VPS37A
membrane
fission
autolysosomal
fusion
lysosome
SNAREs & tethers
EPG5
PLEKHM1
VAMP7/8
HGS/HOPS
RAB7
SNAP29
STX17
lipid
delivery
AAGAB
AP-4
AP4B1
AP4E1
AP4M1
AP4S1
lipid transfer complex
ATG2
ATG9A
TMEM41B
WIPI4
B
fold-change mCh+ cells (9C12WT:Ad5) normalized to NTC condition
10
1
****
NTC
TBK1
VPS37A
ATG3
USP8
TRIM21
C
fold-change mCh+ cells (Ad5 control) normalized to NTC condition
10
1
NTC
ATG16L1
ATG10
PIK3C3
EPG5
ATG101
EI24
ATG9A
AP4E1
TBK1
VPS37A
ATG3
USP8
TRIM21

Figure S3. Ubiquitination and the autophagy pathway are specifically required for viral restriction by TRIM21, related to Figure 2

(A) Full results from the targeted counter-screen. Per-gene significance (indicated by asterisks within bars) was estimated against NTCs by one-way ANOVA with Dunnett's test. We then grouped targets by their origin as hits from the whole-genome screen or hypothesis-driven selections, and tested these overarching groupings against NTCs using a nested one-way ANOVA with BKJ adjustment for false-discovery (q-values annotated on panel).

(B) Stable depletion of TRIM21, UBE2N, ATG10, and ATG101 using CRISPR/Cas9 confirmed via immunoblot.

(C-D) Stable depletion of UBE2N prevented $9C12^{WT}$-directed restriction of Ad5 with (C) similar effect size to depletion of autophagy genes. In TRIM21 KO HEK293T cells, (E) co-depletion of UBE2N or autophagy genes had no additional effect on infection.

(E) Lipidation of LC3 in HEK293T and KO TRIM21 cells, and the effect of Ad5 or $9C12^{WT}$:Ad5 two hours post-infection.

(F) Control infection with Ad5 in panel of cells pre-treated with inhibitors for 2 hours prior to inoculation. Significance was estimated against DMSO treatment for each cell line, using two-way ANOVA and Šídák's test for multiple comparisons. n=2 biological replicates per cell line.

(G) Infection of HEK293T cells pre-treated with MG132 for the indicated durations prior to inoculation with $9C12^{WT}$complexed Ad5.

(H) Panel of cells treated for with the indicated inhibitors at the time of infection with $9C12^{WT}$:Ad5. Inhibitors were removed 1 hour post-infection. Twenty hours later, fluorescent cells were counted, normalized to confluency, and plotted relative to the DMSO control condition. Treatment-wise significance was estimated from n=3 independent replicates by two-way ANOVA corrected for multiple comparisons using Tukey's test.

Fig S3

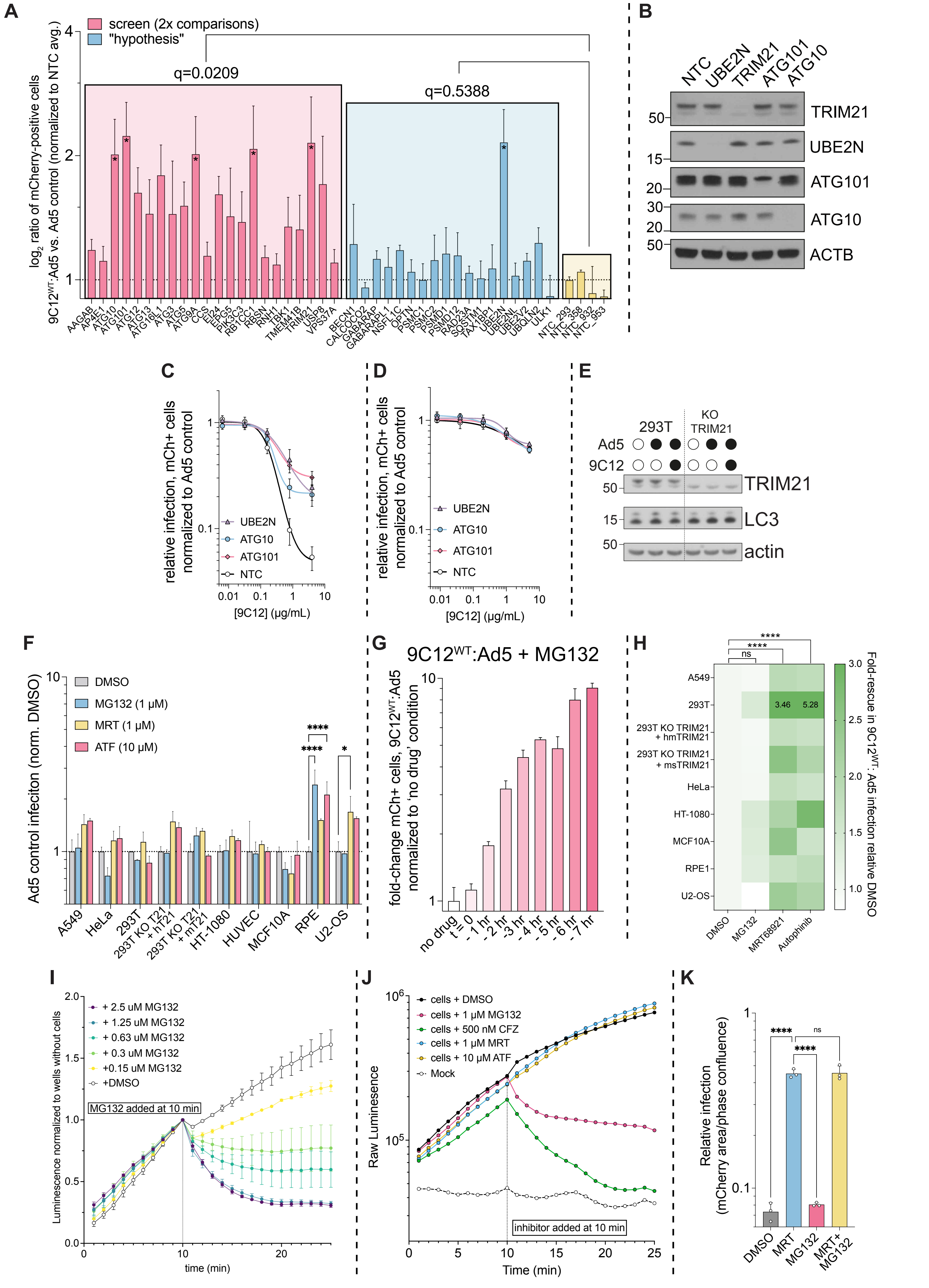

A
screen (2x comparisons)
"hypothesis"
q=0.0209
q=0.5388
9C12WT:Ad5 vs. Ad5 control (normalized to NTC avg.)
log2 ratio of mCherry-positive cells
AAGAB
AP4E1
ATG10
ATG101
ATG12
ATG13
ATG16L1
ATG3
ATG5
ATG9A
CCS
EI24
EPG5
PIK3C3
RB1CC1
RBSN
RNH1
TBK1
TMEM41B
TRIM21
USP8
VPS37A
BECN1
CALCOCO2
GABARAP
GABARAPL1
NSFL1C
OPTN
PSMC1
PSMC2
PSMD1
PSMD12
RAD23A
SQSTM1
TAX1BP1
UBE2N
UBE2NL
UBE2V2
UBQLN2
ULK1
NTC_293
NTC_358
NTC_932
NTC_953
B
NTC
UBE2N
TRIM21
ATG101
ATG10
TRIM21
UBE2N
ATG101
ATG10
ACTB
C
relative infection, mCh+ cells normalized to Ad5 control
UBE2N
ATG10
ATG101
NTC
[9C12] (μg/mL)
D
relative infection, mCh+ cells normalized to Ad5 control
UBE2N
ATG10
ATG101
NTC
[9C12] (μg/mL)
E
293T
KO TRIM21
Ad5
9C12
TRIM21
LC3
actin
F
Ad5 control infeciton (norm. DMSO)
DMSO
MG132 (1 μM)
MRT (1 μM)
ATF (10 μM)
A549
HeLa
293T
293T KO T21 + hT21
293T KO T21 + mT21
HT-1080
HUVEC
MCF10A
RPE
U2-OS
G
9C12WT:Ad5 + MG132
fold-change mCh+ cells, 9C12WT:Ad5 normalized to 'no drug' condition
no drug
t = 0
- 1 hr
- 2 hr
- 3 hr
- 4 hr
- 5 hr
- 6 hr
- 7 hr
H
A549
293T
293T KO TRIM21 + hmTRIM21
293T KO TRIM21 + msTRIM21
HeLa
HT-1080
MCF10A
RPE1
U2-OS
DMSO
MG132
MRT68921
Autophinib
ns
3.46
5.28
Fold-rescue in 9C12WT: Ad5 infection relative DMSO
I
+ 2.5 uM MG132
+ 1.25 uM MG132
+ 0.63 uM MG132
+ 0.3 uM MG132
+0.15 uM MG132
+DMSO
MG132 added at 10 min
Luminescence normalized to wells without cells
time (min)
J
cells + DMSO
cells + 1 μM MG132
cells + 500 nM CFZ
cells + 1 μM MRT
cells + 10 μM ATF
Mock
Raw Luminescence
inhibitor added at 10 min
Time (min)
K
Relative infection (mCherry area/phase confluence)
ns
DMSO
MRT
MG132
MRT+ MG132

Figure S4. TRIM21 physically interacts with antibody-coated virions, related to Figure 2

(A) Expression levels of FR-TRIM21^{WT} and FR-TRIM21^{MUT}.

(B) WT HEK293T, TRIM21 KO and TRIM21 KO cells expressing FR-TRIM21^{WT} or FR-TRIM21^{MUT} were challenged with Ad5 complexed in indicated concentrations of 9C12^{WT}. n=2 biological replicates.

(C) The efficacy of AF647-9C12^{WT} in restricting Ad5 was compared to that of unlabelled 9C12^{WT}. n=2 biological replicates.

(D) Representative confocal image of FR-TRIM21^{MUT} cells inoculated with AF-9C12^{WT}:Ad5, colocalizing with anti-Fab at 30 minutes post-infection.

(E) Representative confocal image of FR-TRIM21^{WT} cells inoculated with AF-9C12^{WT}:Ad5, colocalizing with FK2 at 20 minutes post-infection.

(F) HEK293T cells were electroporated with mRNA encoding the indicated split-luciferase constructs, then infected with Ad5 complexed with 9C12^{WT} or 9C12^{H433A}. Luminescence is reconstituted when the SmBiT and LgBiT components are brought in physical proximity. Data points represent technical replicates from n=2 independent experiments. Significance was estimated by 2-way ANOVA.

Fig S4

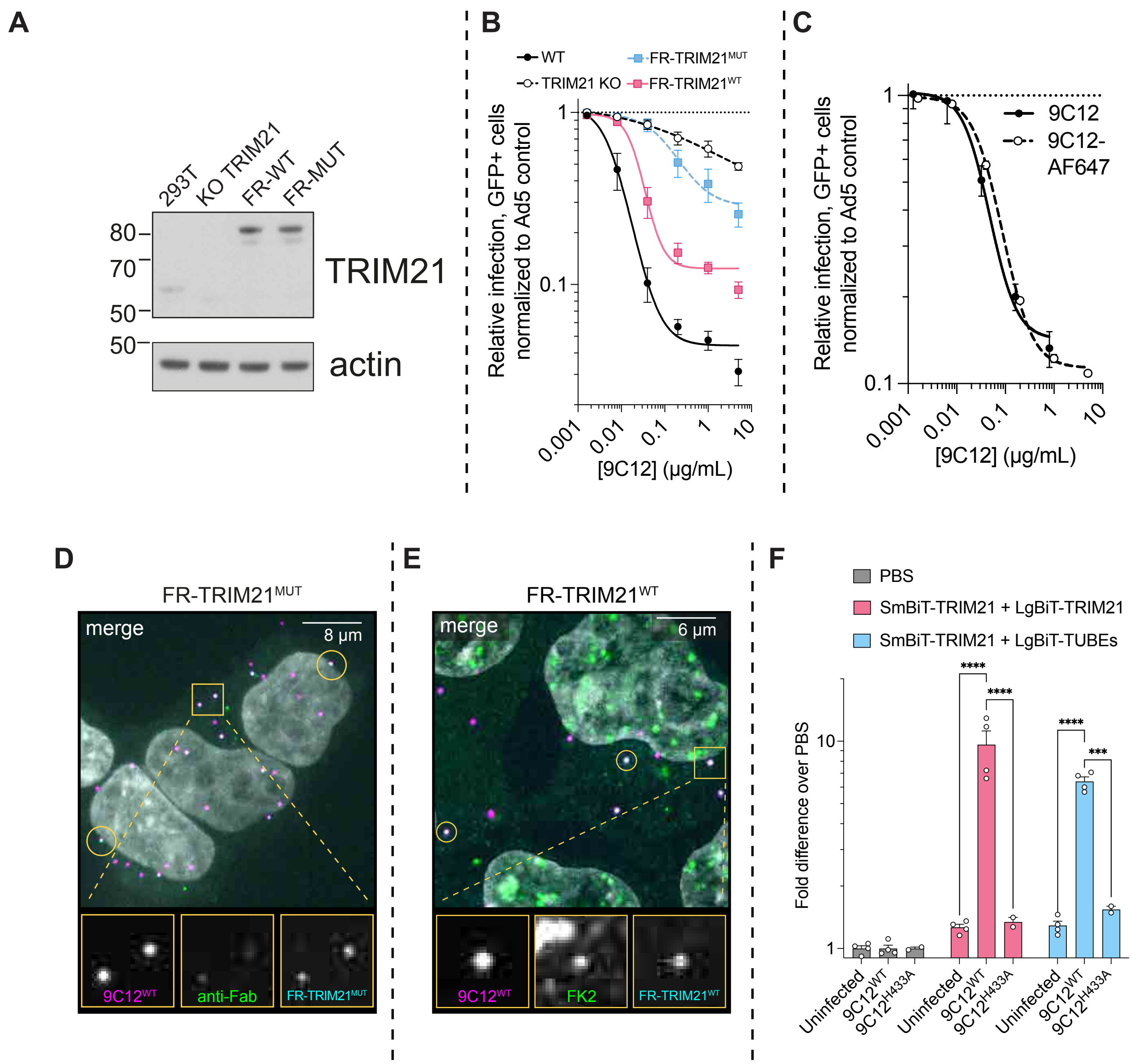
A
293T
KO TRIM21
FR-WT
FR-MUT
80
70
50
50
TRIM21
actin
B
WT
TRIM21 KO
FR-TRIM21MUT
FR-TRIM21WT
Relative infection, GFP+ cells normalized to Ad5 control
[9C12] (μg/mL)
C
9C12
9C12-AF647
Relative infection, GFP+ cells normalized to Ad5 control
[9C12] (μg/mL)
D
FR-TRIM21MUT
merge
8 μm
9C12WT
anti-Fab
FR-TRIM21MUT
E
FR-TRIM21WT
merge
6 μm
9C12WT
FK2
FR-TRIM21WT
F
PBS
SmBiT-TRIM21 + LgBiT-TRIM21
SmBiT-TRIM21 + LgBiT-TUBEs
Fold difference over PBS
Uninfected
9C12WT
9C12H433A
****
***

Figure S5. TRIM21 specifically recruits autophagy factors to antibody-coated viruses, related to Figure 3

(A) Induction of autophagy adaptor expression in HeLa 5KO cells transduced with pTetOne lentiviruses was confirmed by immunoblot 24 hours after doxycycline treatment.

(B) Infectivity of Ad5 without antibodies in HeLa 5KO cells complimented with the indicated autophagy adaptor proteins or control. n=3 independent experiments, analysed by one-way ANOVA and Dunnett's test.

(C) Quantification of colocalization in WT HeLa cells corresponding to the confocal micrograph in **Fig. 3D**. Significance was estimated by two-way ANOVA and Tukey's test from n=4 image fields per condition.

(D) Representative confocal micrographs of colocalization between AF-9C12^{WT}:Ad5, NDP52, and FR-TRIM21^{WT} in HEK293T cells 30 minutes following synchronized infection and treatment with the indicated inhibitors. Corresponds to the quantification in **Fig. 3H**.

(E) Quantification and representative images from structured illumination super-resolution microscopy of LC3-positive compartments engulfing FR-TRIM21 bound 9C12^{WT}:Ad5. Significance was estimated from one experiment with n=7 image fields per condition by 2-way ANOVA.

Fig S5

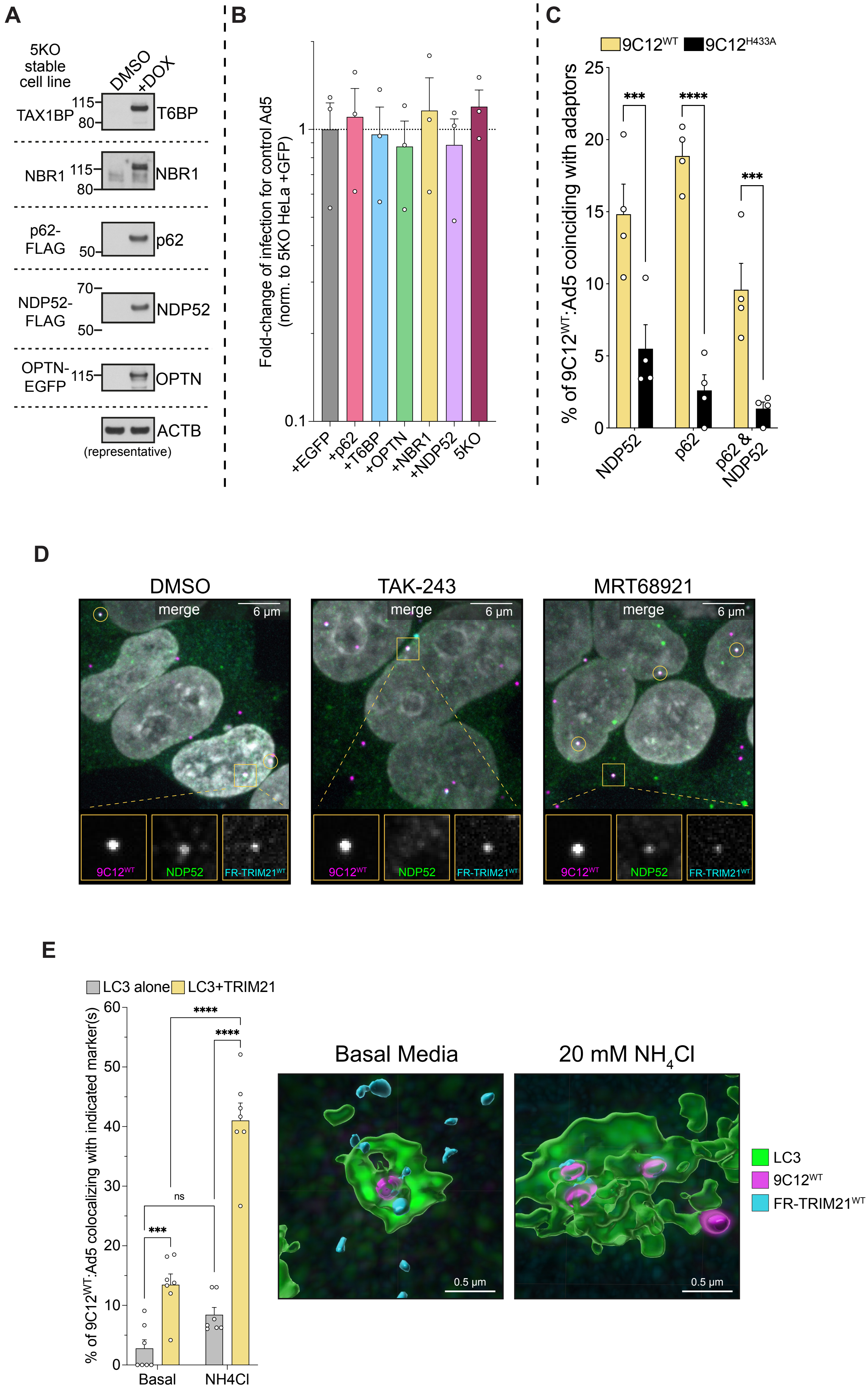
A
5KO stable cell line
DMSO
+DOX
TAX1BP
115
80
T6BP
NBR1
115
80
NBR1
p62-FLAG
50
p62
NDP52-FLAG
70
50
NDP52
OPTN-EGFP
115
OPTN
ACTB
(representative)
B
Fold-change of infection for control Ad5 (norm. to 5KO HeLa +GFP)
1
0.1
+EGFP
+p62
+T6BP
+OPTN
+NBR1
+NDP52
5KO
C
9C12WT
9C12H433A
% of 9C12WT:Ad5 coinciding with adaptors
25
20
15
10
5
0
***
****
***
NDP52
p62
p62 & NDP52
D
DMSO
TAK-243
MRT68921
merge
6 µm
9C12WT
NDP52
FR-TRIM21WT
E
LC3 alone
LC3+TRIM21
% of 9C12WT:Ad5 colocalizing with indicated marker(s)
60
50
40
30
20
10
0
****
****
ns
***
Basal
NH4Cl
Basal Media
20 mM NH4Cl
0.5 µm
LC3
9C12WT
FR-TRIM21WT

Figure S6. Antibody-directed xenophagy degrades the adenovirus capsid, related to figure 3

(A) Extended data from **Fig. 3J**. Per-cell quantification of colocalization between FR-TRIM21 mutants and AF-9C12^{WT}:Ad5 at intervals following infection. Significance was estimated using two-way ANOVA and Tukey's test. Quantifications were from n=5 image fields per condition.

(B) Representative confocal micrograph obtained in live HEK293T 60 minutes following synchronized infection. EGFP-LC3B staining was lost on compartments that stained positive for LysoTracker, and this was accompanied by a marked change in the shape and intensity of FR-TRIM21 and AF-9C12^{WT}:Ad5 puncta.

(C) Representative confocal micrographs showing incarceration of 9C12^{WT}- and FR-TRIM21^{WT}-positive Ad5 in large LAMP1-positive lysosomes with visible lumens.

(D) HEK293T cells expressing EGFP-FR^{MQV} fused to a nanobody against the Fab domain of human IgG1 were infected with Ad5 coated with the indicated antibody mutants. Conversion of red+green to red-only signal is consistent with delivery of 9C12^{WT}:Ad5 to acidic lysosomes. Data are from n>5 image fields per condition, and significance was estimated by two-way ANOVA.

(E) Immunoblot for LC3, TRIM21, and adenovirus proteins (polyclonal anti-Ad5) two hours after infection with the indicated virus complexes and inhibitors. Drug concentrations were 1 μM MG132 and 400 nM BafA1.

(F) Immunoblot for TRIM21 and adenovirus proteins 48 hours after infection with the indicated virus complexes and inhibitors. Drug concentrations were 1 μM MG132 and 1 μM MRT68921.

Fig S6

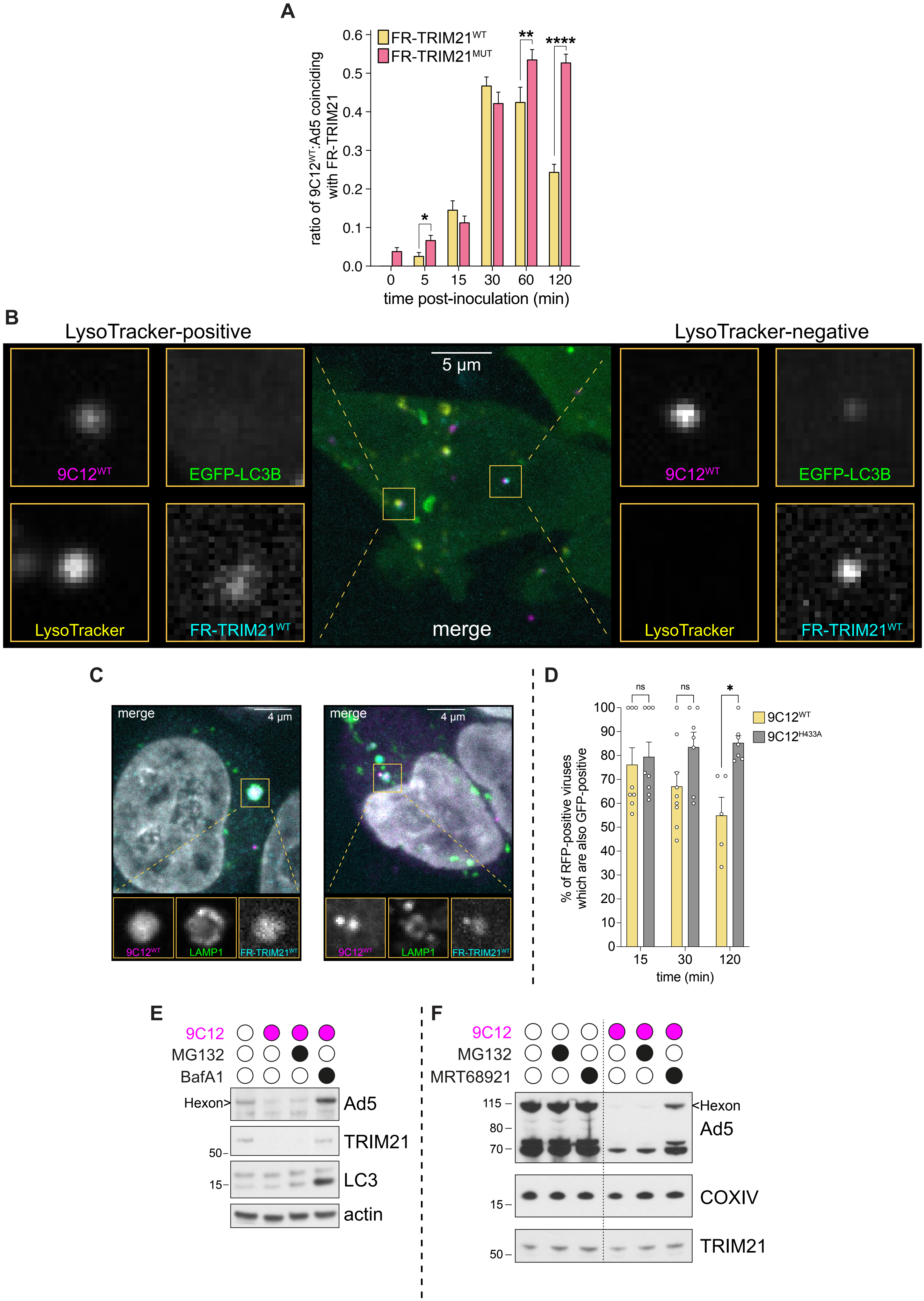

A
ratio of 9C12WT:Ad5 coinciding with FR-TRIM21
FR-TRIM21WT
FR-TRIM21MUT
0.0
0.1
0.2
0.3
0.4
0.5
0.6
*
**
****
0
5
15
30
60
120
time post-inoculation (min)
B
LysoTracker-positive
LysoTracker-negative
9C12WT
EGFP-LC3B
LysoTracker
FR-TRIM21WT
5 µm
merge
C
merge
4 µm
9C12WT
LAMP1
FR-TRIM21WT
D
% of RFP-positive viruses which are also GFP-positive
ns
ns
*
9C12WT
9C12H433A
15
30
120
time (min)
E
9C12
MG132
BafA1
Hexon>
Ad5
TRIM21
50
LC3
15
actin
F
9C12
MG132
MRT68921
115
80
70
<Hexon
Ad5
COXIV
15
TRIM21
50

Figure S7. Antibody-mediated restriction of *Salmonella* is separable from entry-blocking and requires TRIM21, related to Figure 4

(A) Data correspond to **Fig. 4A-B**. The mean integrated FK2 intensity was calculated from thousands of individual FK2-positive cytosolic bacteria and normalized to control conditions. Significance was estimated by two-way ANOVA and Tukey's test for multiple comparisons. FK2 intensity from each bacteria was individually quantified from n=20 image fields per condition.

(B-C) *S.* Typhimurium colocalizing with electroporated fluorescent anti-*Salmonella* antibodies in FR-$TRIM21^{WT}$ MEFs. (B) Representative confocal images 1 hour post-infection are presented with corresponding fluorescence intensity in each channel plotted along a line profile bisecting selected bacteria. (C) Proportion of bacteria which colocalized with electroporated antibodies that were also FK2+. Significance was estimated from n=24-25 fields per condition by unpaired two-tailed T-test.

(D) Example segmentation of hyper-proliferating mCherry *S.* Typhimurium in the cytosol of infected MEFs.

(E) Real-time increase in raw fluorescent intensity of segmented MEFs infected with mCherry *S.* Typhimurium.

(F) The antiproliferative effect of anti-Salmonella or control antibodies, from n=3 experiments.

(G) Anti-*Salmonella* antibodies prevented cell penetration by *S.* Typhimurium, as measured by the normalized count of fluorescent bacteria in cells at early timepoints, from n=3 experiments.

(H) Effect of titration of the inoculum density by dilution of untreated *S.* Typhimurium on the rate of cytosolic hyper-proliferation, from n=3 independent experiments.

Fig S7

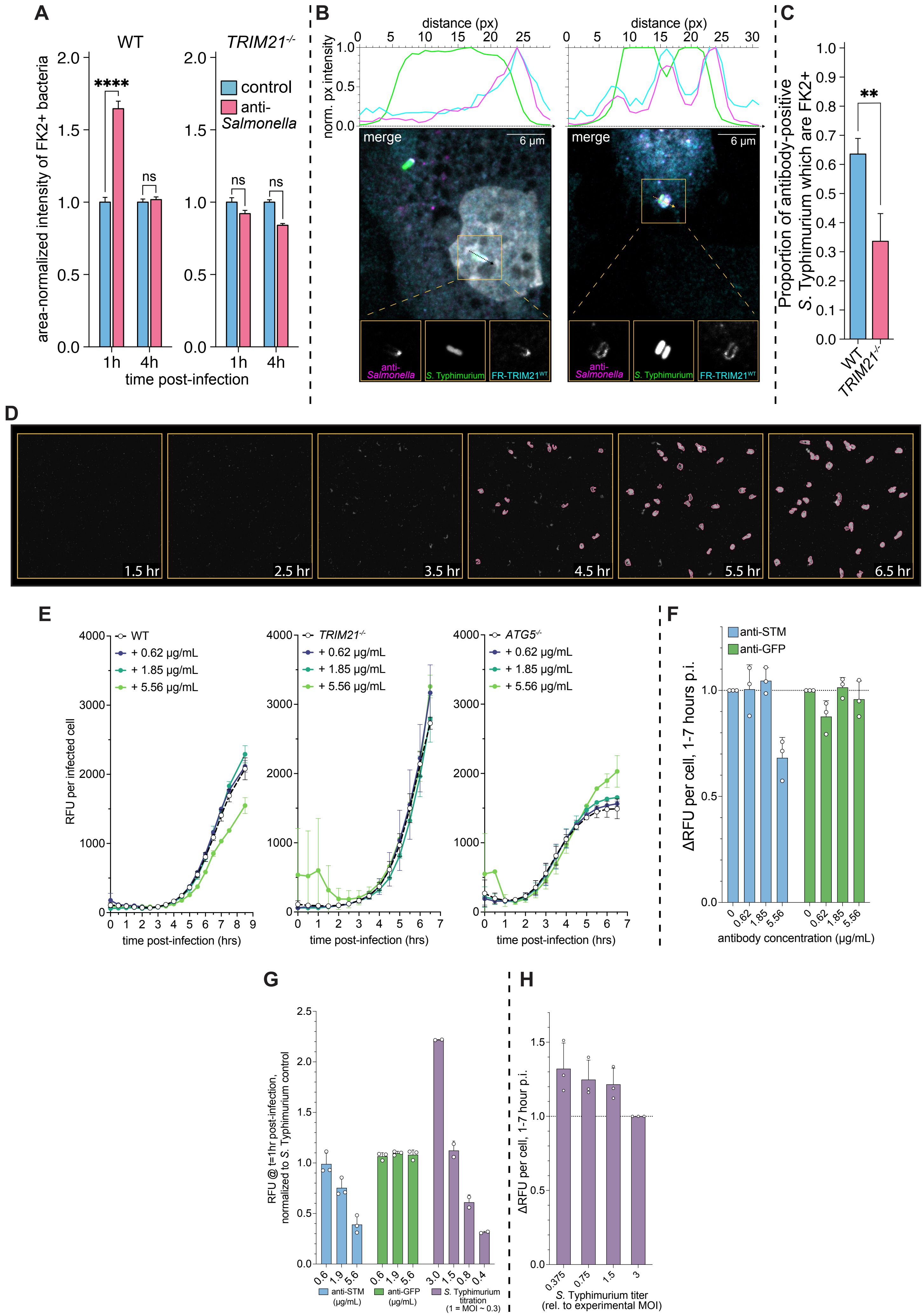
A
WT
TRIM21-/-
area-normalized intensity of FK2+ bacteria
****
ns
ns
ns
control
anti-Salmonella
1h
4h
time post-infection
B
distance (px)
norm. px intensity
merge
6 μm
anti-Salmonella
S. Typhimurium
FR-TRIM21WT
C
Proportion of antibody-positive S. Typhimurium which are FK2+
**
WT
TRIM21-/-
D
1.5 hr
2.5 hr
3.5 hr
4.5 hr
5.5 hr
6.5 hr
E
WT
TRIM21-/-
ATG5-/-
+ 0.62 μg/mL
+ 1.85 μg/mL
+ 5.56 μg/mL
RFU per infected cell
time post-infection (hrs)
F
anti-STM
anti-GFP
ΔRFU per cell, 1-7 hours p.i.
antibody concentration (μg/mL)
G
RFU @ t=1hr post-infection, normalized to S. Typhimurium control
anti-STM (μg/mL)
anti-GFP (μg/mL)
S. Typhimurium titration (1 = MOI ~ 0.3)
H
ΔRFU per cell, 1-7 hour p.i.
S. Typhimurium titer (rel. to experimental MOI)

Figure S8. ADX is responsible for TRIM21-mediated restriction of *S.* Typhimurium, related to Figure 4

(A) The rate of *S.* Typhimurium cytosolic hyper-proliferation in the presence of anti-*Salmonella* antibodies was rescued by TRIM21 knockout in *RNF213*$^{-/-}$ MEFs. Significance was estimated from n=3 experiments by two-way ANOVA with Šídák's test for multiple comparisons.

(B) TRIM21 clustering around antibody-coated *S.* Typhimurium. LgBit-TRIM21 and SmBit-TRIM21 were purified from *E.* coli and mixed with IgG against lipopolysaccharide. Luciferase signal was measured in the presence or absence of *Salmonella.* Significance was estimated from n=3 experiments by two-way ANOVA and Šídák's test for multiple comparisons.

(C) Colocalization between *S*. Typhimurium and p62 in MEF *RNF213*$^{-/-}$ and MEF *RNF213*$^{-/-}$ *TRIM21*$^{-/-}$ cells. Significance was estimated from n=20 image fields per condition by two-way ANOVA.

(D) Immunoblot for p62 and LC3 in WT MEF cells infected with *S*. Typhimurium and antibodies.

(E) Super-resolution microscopy of LC3 in MEFs infected with antibody-coated *S*. Typhimurium. Quantifications were from one experiment with n=7 image fields per condition, and significance was estimated by two-way ANOVA.

(F) Results from qPCR of *S.* Typhimurium genomic DNA in tissues collected from animal subjects in the passive serum transfer experiment. Significance was estimated by two-way ANOVA and Dunnett's test for multiple comparisons. There were n=6 mice per group.

Fig S8

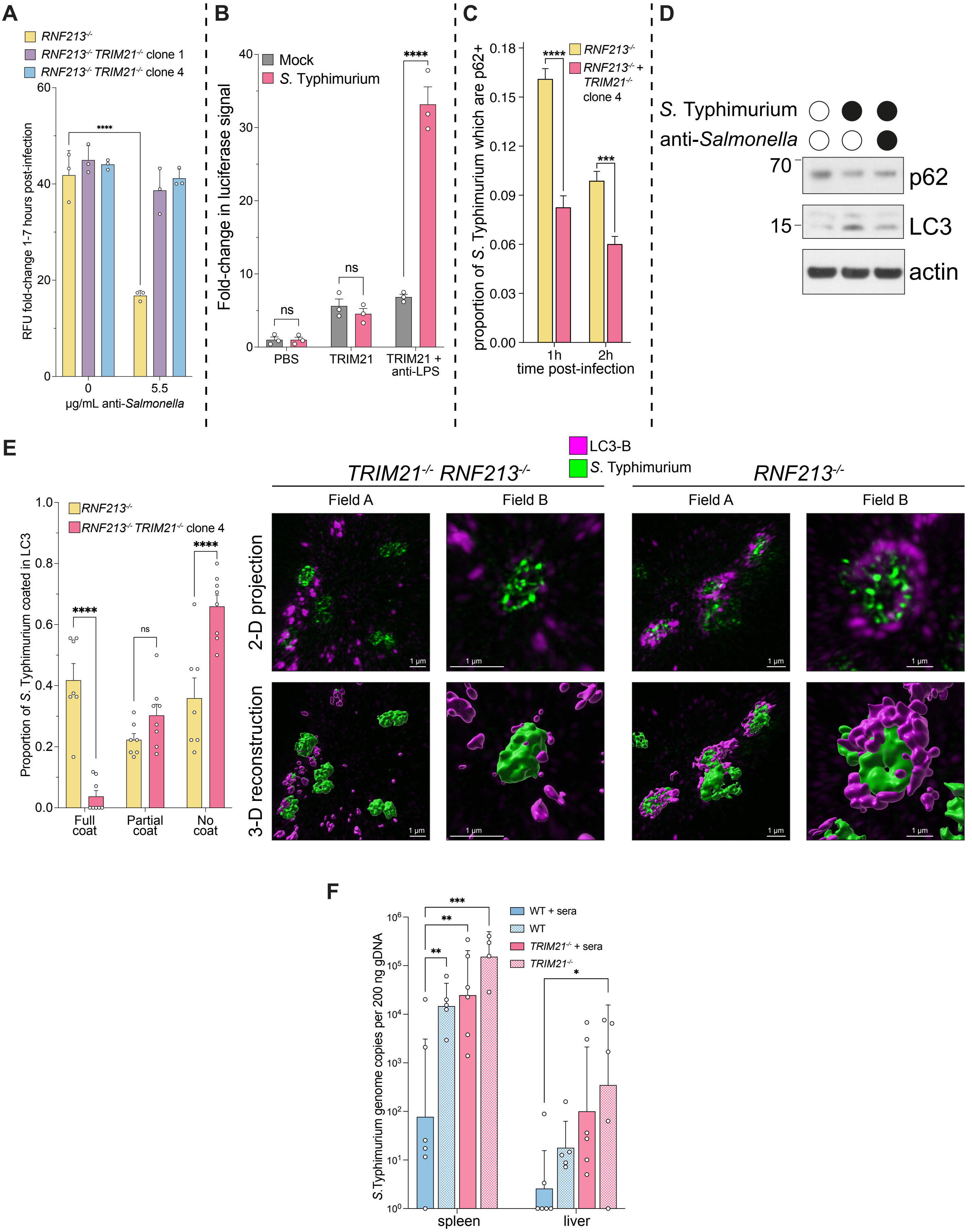
A
RNF213-/-
RNF213-/- TRIM21-/- clone 1
RNF213-/- TRIM21-/- clone 4
RFU fold-change 1-7 hours post-infection
60
40
20
0
****
0
5.5
μg/mL anti-Salmonella
B
Mock
S. Typhimurium
Fold-change in luciferase signal
40
30
20
10
0
ns
ns
****
PBS
TRIM21
TRIM21 + anti-LPS
C
RNF213-/-
RNF213-/- + TRIM21-/- clone 4
proportion of S. Typhimurium which are p62+
0.18
0.15
0.12
0.09
0.06
0.03
0.00
****
***
1h
2h
time post-infection
D
S. Typhimurium
anti-Salmonella
70
15
p62
LC3
actin
E
LC3-B
S. Typhimurium
TRIM21-/- RNF213-/-
RNF213-/-
Field A
Field B
Field A
Field B
2-D projection
3-D reconstruction
1 μm
RNF213-/-
RNF213-/- TRIM21-/- clone 4
Proportion of S. Typhimurium coated in LC3
1.0
0.8
0.6
0.4
0.2
0.0
****
ns
****
Full coat
Partial coat
No coat
F
WT + sera
WT
TRIM21-/- + sera
TRIM21-/-
S.Typhimurium genome copies per 200 ng gDNA
10^6
10^5
10^4
10^3
10^2
10^1
10^0
***
**
**
*
spleen
liver
